# Impact of a recent cancer diagnosis on bleeding risk after myocardial infarction

**DOI:** 10.1136/heartjnl-2025-326524

**Published:** 2025-12-30

**Authors:** Kok Weng Ow, Freya Tyrer, Freek Van Den Berg, Jennifer Lai, Sally Vernon, Lizz Paley, Florian A Wenzl, Clive Weston, Mark J Rutherford, Paul C Lambert, Mark A de Belder, John Deanfield, Michael D Peake, David Adlam

**Affiliations:** 1 Department of Cardiovascular Sciences and NIHR Leicester Biomedical Research Centre, University of Leicester, Leicester, UK; 2 National Disease Registration Service, NHS England, London, UK; 3 Department of Population Health Sciences, University of Leicester, Leicester, UK; 4 Observational and Pragmatic Research Institute, England, UK; 5 Department of Experimental Cardiology, University Medical Centre Groningen, Groningen, The Netherlands; 6 Center for Molecular Cardiology, University of Zurich, Zürich, Switzerland; 7 Department of Clinical Sciences, Karolinska Institutet, Stockholm, Sweden; 8 Glangwili General Hospital, Carmarthen, UK; 9 Cancer Registry of Norway, Norwegian Institute of Public Health, Oslo, Norway; 10 Department of Medical Epidemiology and Biostatistics, Karolinska Institutet, Stockholm, Sweden; 11 National Institute for Cardiovascular Outcomes Research (NICOR), NHS Arden and Greater East Midlands Commissioning Support Unit, Leicester, UK; 12 William Harvey Research Institute, Queen Mary University of London, London, UK; 13 Institute of Cardiovascular Science, University College London, London, UK; 14 National Institute of Cardiovascular Outcomes Research (NICOR), London, UK; 15 University of Leicester, Leicester, UK; 16 Leicester British Heart Foundation Centre of Research Excellence, Leicester, UK

**Keywords:** Myocardial Infarction, Acute Coronary Syndrome

## Abstract

**Background:**

Determining the most appropriate treatment in patients with cancer with an acute myocardial infarction (MI) can be challenging. Optimal management requires an understanding of bleeding risk which may be different in this population. This study aimed to investigate the bleeding risk among patients with MI with and without cancer, in the first and second year post-MI.

**Methods:**

Patients with MI, with and without cancer were identified from a national cardio-oncology database from England between 2006 and 2019. The outcome was a presentation to hospital for a major bleeding event, with patients followed for a maximum of 24 months. Inverse probability weighting was used to compare cancer and non-cancer cohorts in time-to-event analyses.

**Results:**

587 279 patients with MI were identified, 9820 (1.7%) had cancer and 577 459 (98.3%) did not. Colorectal, prostate, breast, lung and bladder cancer were the most common types of cancer. The rate of hospital presentation for bleeding in the first year post-MI was higher in patients with cancer than the non-cancer reference population (HR: 1.53, 95% CI 1.45 to 1.62, p<0.001). 506 280 patients with MI were followed up in the second year post-MI. 5666 (1.1%) had cancer and 500 614 (98.9%) did not. The bleeding rate in patients with MI with cancer remained elevated in the second year post-MI (HR: 1.42, 95% CI 1.29 to 1.56, p<0.001). There were marked differences in bleeding between cancer types.

**Conclusion:**

In this real-world observational study, patients with cancer had an increased bleeding risk in the first year post-MI which decreased but persisted in the second year after MI. Bleeding risk in patients with cancer must be carefully assessed post-MI.

**WHAT IS ALREADY KNOWN ON THIS TOPIC**

- Although active cancer is recognised as a risk factor for increased bleeding, the specific bleeding risks associated with different types of cancer and MI in patients with cancer post-MI remain unclear. It is also unclear if the bleeding risk remains static or changes with time.

**WHAT THIS STUDY ADDS**

- Patients with recently diagnosed cancer are observed to be at an increased risk of serious bleeding requiring hospitalisation compared with patients without cancer post-MI, which persists beyond the 1-year post-MI period, although lower compared with the first year post-MI. Cancer type is an important factor to be considered when estimating bleeding risk in patients with cancer post-MI.

**HOW THIS STUDY MIGHT AFFECT RESEARCH, PRACTICE OR POLICY**

- Current guidelines still do not clearly discriminate between the different cancer types, and further work is needed to identify optimal personalised treatment strategies, which favourably balance the ischaemic and bleeding risk in this population.

## Introduction

 Cardiovascular disease (CVD) and cancer are common. They share common risk factors,^1^ notably obesity,^2^ smoking^3^ and diabetes,^4^ and while conventionally managed by separate specialty teams, therapeutic advancements have resulted in a growing population of patients living with both conditions.^5 6^ Despite this increasing co-prevalence, there remains a paucity of high-quality evidence to guide the optimal management of myocardial infarction (MI) in patients with cancer. Patients with cancer are often excluded from major interventional^7^ and pharmacological trials in MI,^8^ and cancer has not been incorporated as a covariate in contemporary bleeding risk prediction models for patients receiving dual antiplatelet therapy (DAPT).^9 10^

The risk of MI is heightened in patients with cancer.^11^ MI represents approximately 20% of all CVD admissions in patients with cancer^12^ and up to 10% of patients who present with MI have current or prior history of cancer.^13^

Bleeding remains a major concern in patients with cancer. Despite being at an increased risk of ischaemic cardiovascular events, patients with cancer are also paradoxically susceptible to bleeding events,^14^ either as a direct complication of the malignancy itself or secondary to cancer treatment.^15 16^ In patients with cancer with concomitant venous thromboembolism on anticoagulation, bleeding has been shown to be a significant cause of mortality.^17^

Managing ischaemic and bleeding risks is challenging in patients with MI and cancer because these risks can vary between the different malignancies.^18^ This results in difficulty when balancing the risks and benefits of administering antiplatelet therapies to optimise the risk profile for a patient with cancer compared with patients without cancer. International guidelines recommend tailoring decisions on invasive management, antiplatelet type and duration, based on personalised bleeding risk.^19 20^

The urgent unmet clinical need in this complex cohort of patients highlights the importance of high-quality observational data required to explore the impact of cancer on the bleeding risk in patients with MI who would be treated with antiplatelets. We therefore used the Virtual Cardio-Oncology Research Initiative (VICORI) database to investigate (1) the risk of bleeding among patients with MI with recent cancer compared with patients without cancer, in the first year post-MI, stratified by the type of MI, coronary stenting and cancer type and (2) the risk of bleeding among patients with MI with recent cancer compared with patients without cancer, in the second year post-MI, by when patients would be expected to be on single antiplatelet therapy (SAPT), stratified by the type of MI, coronary stenting and cancer type. We focused on patients who were not receiving anticoagulation for any indications to concentrate on bleeding risk relating to guideline-based standard of care treatment with antiplatelets after MI.

## Methods

### Data sources

This study used data from the VICORI database,^21^ which captures adults who were hospitalised for a cardiac cause, underwent a cardiac intervention and who have a cancer diagnosis, from routinely collected data.^21^ Based in England, it links data from: (1) National Cancer Registration Dataset (NCRD),^22^ (2) CVD audits from the National Institute for Cardiovascular Outcomes Research, specifically used in this study were the Myocardial Infarction National Audit Project (MINAP)^23^ and National Audit for Percutaneous Coronary Intervention (NAPCI),^24^ (3) Hospital Episode Statistics (HES) for hospital admission data^25^ and (4) Office for National Statistics death registry using National Health Service (NHS) numbers. MINAP includes data on patients with MI, while NAPCI collects data on patients who underwent percutaneous coronary intervention (PCI). This work uses data that has been provided by patients and collected by the NHS as part of their care and support. The data are collated, maintained and quality assured by the National Disease Registration Service, part of NHS England.^26 27^

### Study design and study population

This is a retrospective observational cohort study where the VICORI database was used to identify patients with MI between 1 January 2006 to 31 March 2019. All MI events were initially extracted, acknowledging that individual patients may experience one or multiple events. For patients with an MI diagnosis occurring between 2006 and 2019, only the first recorded MI within this period was retained for analysis. Subsequent MI events within this time frame were categorised as ‘Not first MI event in window of interest’. Patients with a documented MI prior to 2006 who subsequently experienced an MI between 2006 and 2019 were excluded (figure 1). Patients either had an ST-elevation MI (STEMI) or non-STEMI (NSTEMI) using published definitions.^28^ Patients were also included if they underwent PCI as part of their MI treatment, identified in NAPCI. Duplicate and overlapping records between MINAP and NAPCI were removed. While information from NAPCI was prioritised, the best available information was obtained between the datasets.

**Figure 1:**
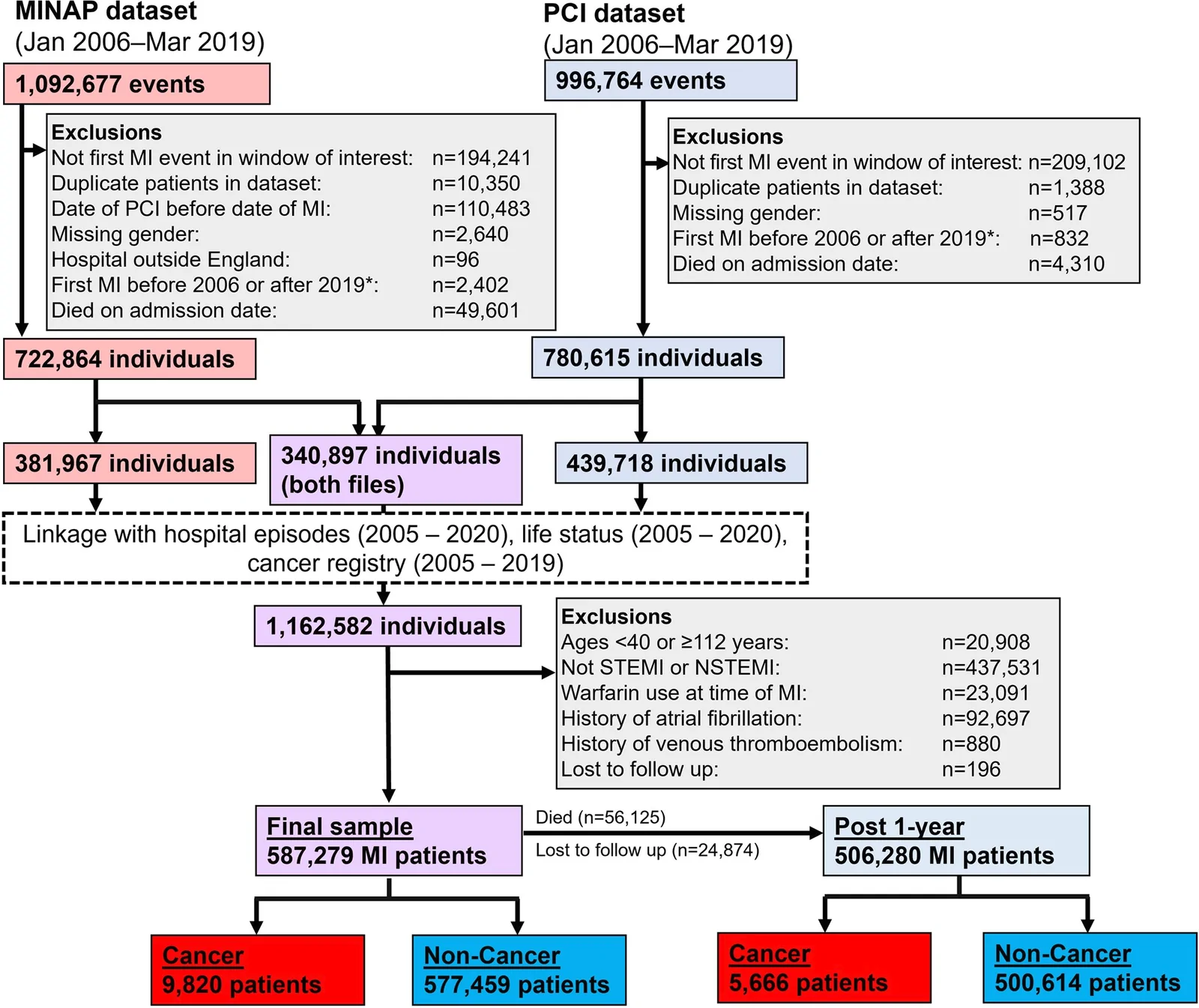
Flowchart illustrating the data sources and patient identification for the study cohort. Sequential exclusion criteria were applied, with the number of individuals excluded at each step shown within the corresponding box. The final sample represents the cohort remaining after all exclusions were applied. *To maintain confidentiality, the provision of dates by NICOR was randomly changed to within 7 days either side of the ‘true’ date (in the same direction for each patient). For consistency, and to correctly identify previous cancers, previous exposures and subsequent outcomes, we corrected these dates where we had additional information and excluded patients if the dates fell outside of the window of interest. MI, myocardial infarction; MINAP, Myocardial Infarction National Audit Project; NICOR, National Institute for Cardiovascular Outcomes Research; NSTEMI, non-STEMI; PCI, percutaneous coronary intervention; STEMI, ST-elevation MI.

Patients with MI were identified if they had a recent cancer diagnosis, defined as a new diagnosis of cancer in the 1 year preceding the MI, recorded in NCRD. Each tumour is coded with a specific site, morphology and behaviour classification which enables mapping to other major coding classification systems, including International Classification of Diseases, 10th Revision (ICD-10).^22^ Only malignant cancers were included, defined using ICD-10 codes C00–C97 (excluding C44/non-melanoma cancers)^29^ (online supplemental table 1).

Exclusion criteria were missing sex, MI occurring outside the time-window of interest, date of PCI before date of MI, coexisting venous thromboembolism, coexisting atrial fibrillation, concomitant warfarin use and a non-MI diagnosis. The study focuses on estimating bleeding risk associated with conventional post-MI antiplatelet use. To avoid confounding, patients who have an indication for anticoagulation which may lead to the use of triple therapy or lifelong anticoagulation post-MI were excluded. Numbers excluded are demonstrated in figure 1.

Covariates include age (continuous variable), sex (male/female), ethnicity (categorical variable, white, South Asian, black, other, not known), geographical region (East Midlands, East of England, London, North East, North West, South East, South West, West Midlands, Yorkshire and the Humber), body mass index (categorical variable, underweight, normal, overweight, obese, very obese, missing), hypertension (categorical, yes, no, not known), hypercholesterolaemia (categorical, yes, no, not known), diabetes (categorical, yes, no, not known), falls and fractures (binary variable, yes/no), previous bleeding (binary variable, yes/no), renal disease (binary variable, yes/no), cerebrovascular disease (categorical, yes, no, not known), smoking (categorical variable, people who smoke, people who have never smoked, people who have quit smoking, missing). Ethnicity, previous bleeding, falls and fractures were obtained from HES, while remaining covariates were obtained from either NAPCI or MINAP.

To define the study population in the second year post-MI, the follow-up period was set from months 12–24 after MI. For this component of the analysis, patients would need to have survived the first 12 months and still had follow-up information (with a ‘last known status’ date after the initial 12-month follow-up period) to be included. Patients who did not die but had a ‘last known status’ date earlier than the period of interest were treated as being lost to follow-up.

### Study outcomes

The outcome of interest was a presentation to hospital for a major bleeding event. Patients were initially followed up for 12 months post-MI, where bleeding events were identified from the HES dataset using ICD-10 codes (primary diagnosis in the first position), modified from the literature^30^ to include iron deficiency anaemia (online supplemental table 2). Only the first bleeding event is counted, and follow-up was stopped after this first event.

Patients who lived past year 1 were further followed from months 12–24 after the index MI. Relative bleeding risk in this population was conditional on surviving or having information beyond the first 12-month period. Subgroup analyses were performed where the results were stratified by type of MI, coronary stenting and cancer type.

### Statistical analysis

Continuous variables are expressed as mean and SD. Categorical variables are expressed using numbers and percentages. Patient demographics and clinical variables for patients with MI with and without cancer were compared using means or proportions, as relevant, and the standardised mean difference was used to assess imbalance between covariates.

We used propensity score methodology, specifically inverse probability weighting, to mitigate selection bias and to balance cohort characteristics prior to study entry. This allows us to control for the effects of confounding and ensure balance between the cancer and non-cancer groups at index. Logistic regression using cancer as the outcome was used to estimate propensity scores based on selected covariates. A directed acyclic graph (online supplemental figure 1) was constructed to identify covariates required to calculate the propensity scores, based on previous literature.^31–34^ Covariates selected were age, sex, hypercholesterolaemia, hypertension, smoking, falls and fractures, previous history of bleeding, renal disease and diabetes. Missing covariates were included in the generation of propensity scores as a separate category. Continuous variables were modelled as linear covariates. Inverse probability weighting based on propensity scores was used to produce a balanced cohort of patients with and without cancer. Weights were stabilised by multiplying the inverse probability weighting by the marginal probability of having cancer to allow for very high and very low propensity scores.^35 36^ Standardised mean difference (SMD) of <0.1 for each variable was used to assess if balance had been achieved between the cohorts.^37^ Cox proportional hazard modelling and flexible parametric models^38^ were used to investigate the impact of cancer on bleeding outcomes following MI. Flexible parametric models model the log cumulative baseline hazard using restricted cubic splines. All time-to-event analyses used the weighted sample. Inverse probability weighting was retained when analysing the different types of MI and if patients underwent coronary stenting. These models were fit with cancer as the exposure measure with 5 df for the spline function to flexibly model the cumulative baseline risk of bleeding. We further relaxed the proportional hazard assumption for the effect of prior cancer on bleeding by introducing a time-dependent effect with 3 df (knots placed at the 33rd and 66th percentile). The flexible parametric modelling approach is similar to the Cox model, while directly estimating absolute risks in both groups, smoothly across follow-up time. The use of the parametric modelling also allowed the quantification of uncertainty as a smooth function over time since MI.

Further subgroup analyses were performed on common cancers. Propensity scores and inverse probability weights were regenerated when analysing different types of cancer, comparing specific cancer types to patients without cancer. These models were fitted instead with the specific cancer type as the exposure measure, again, with 5 df for the spline function for the (log) cumulative baseline hazard, bleeding as the outcome and the time-dependent effects of the exposure also taken into account with a simpler spline function for the timescale with 3 df for that interaction term. Patients who have multiple cancer diagnoses were included in each cancer type. When analysing prostate cancer, female sex was excluded, whereas male sex was excluded when analysing breast cancer.

Sensitivity analysis was performed by excluding patients who had a history of bleeding within 5 years of the index MI. As iron deficiency anaemia could be considered a symptom of bleeding, we also conducted a sensitivity analysis by removing it from our definition. We used bootstrapping to calculate CIs (robust variance sandwich estimators) to account for the variability of the propensity scores used to derive weights. This study is reported in accordance with the STROBE (Strengthening the Reporting of Observational Studies in Epidemiology) guidelines.^39^ Analyses were performed using Stata/SE V.17 (StataCorp, College Station, Texas, USA). Stata package ‘stpm2’ was used to examine the rate for bleeding.^40^

### Patient and public involvement

VICORI has a cardiovascular Patient and Public Involvement and Engagement (PPIE) group who were involved in all stages of the research presented. Our PPIE group co-developed the concept of VICORI and supported the original funding applications to Cancer Research UK and British Heart Foundation, in addition to overseeing the research progression and the results presented in this manuscript. Going forward, they will support the development of a plain English summary of the research findings to be shared on social media, including publication on the VICORI website: https://vicori.le.ac.uk/patients-and-public/.

## Results

1 162 582 patients were identified between 2006 and 2019 (figure 1). 587 279 eligible patients with MI were included in the analysis where they were first followed up for 1 year for a bleeding event requiring hospital presentation.

Unweighted baseline characteristics of patients with and without cancer are presented in table 1 and online supplemental table 3 for the first and second years post-MI, respectively. Patients with cancer were older (mean (SD) age, 73.1 (10.3) years; 6813 (69.4%) male), more likely to have hypertension, diabetes, previous bleeding, previous falls and fractures, renal disease and cerebrovascular disease compared with patients without cancer (67.1 (13.1) years; 391 704 (67.8%) male).

**Table 1:** Unweighted baseline characteristics of the study population in the first year after myocardial infarction

Characteristics	Non-cancer/comparison cohort		Cancer cohort		Difference in proportions/means between groups	Standardised mean difference
	N (%)/mean (SD)		N (%)/mean (SD)			
Total	577 459	100	9820	100	–	–
Age	67.05	13.1	73.13	10.3	6.08	0.516
Gender						
Male	391 704	67.83	6813	69.38	1.55	0.033
Female	185 755	32.17	3007	30.62	−1.55	−0.033
Ethnicity						
White	503 206	87.14	9045	92.11	4.97	0.163
Asian	32 610	5.65	281	2.86	−2.79	−0.138
Black	5359	0.93	87	0.89	−0.04	−0.004
Other	24 106	4.17	277	2.82	−1.35	−0.074
Not known	12 178	2.11	130	1.32	−0.79	−0.06
Geographical region						
East Midlands	41 052	7.11	637	6.49	−0.62	−0.025
East of England	60 418	10.46	1165	11.86	1.4	0.044
London	76 271	13.21	1148	11.69	−1.52	−0.046
North East	41 433	7.18	614	6.25	−0.93	−0.037
North West	84 595	14.65	1558	15.87	1.22	0.034
South East	78 754	13.64	1369	13.94	0.3	0.009
South West	63 418	10.98	1250	12.73	1.75	0.054
West Midlands	67 801	11.74	947	9.64	−2.1	−0.068
Yorkshire and the Humber	63 717	11.03	1132	11.53	0.5	0.016
BMI category						
Underweight	6518	1.13	217	2.21	1.08	0.084
Normal	103 589	17.94	1980	20.16	2.22	0.057
Overweight	139 536	24.16	1929	19.64	−4.52	−0.109
Obese	87 711	15.19	929	9.46	−5.73	−0.175
Very obese	9320	1.61	81	0.82	−0.79	−0.072
Missing	230 785	39.97	4684	47.7	7.73	0.156
Hypertension						
No	286 443	49.6	4598	46.82	−2.78	−0.056
Yes	269 473	46.67	4757	48.44	1.77	0.036
Unknown	21 543	3.73	465	4.74	1.01	0.05
Hypercholesterolaemia						
No	293 730	50.87	5164	52.59	1.72	0.034
Yes	262 053	45.38	4167	42.43	−2.95	−0.059
Unknown	21 676	3.75	489	4.98	1.23	0.06
Diabetes						
No	444 756	77.02	7336	74.7	−2.32	−0.054
Yes	124 193	21.51	2291	23.33	1.82	0.044
Unknown	8510	1.47	193	1.97	0.5	0.038
Falls and fractures						
No	538 667	93.28	9030	91.96	−1.32	−0.051
Yes	38 792	6.72	790	8.04	1.32	0.051
Previous bleeding						
No	522 687	90.51	7661	78.01	−12.5	−0.348
Yes	54 772	9.49	2159	21.99	12.5	0.348
Renal disease						
No	511 466	88.57	7619	77.59	−10.98	−0.296
Yes	65 993	11.43	2201	22.41	10.98	0.296
Smoking						
People who smoke	207 186	35.88	2817	28.69	−7.19	−0.154
People who have never smoked	188 951	32.72	3646	37.13	4.41	0.093
People who have quit smoking	150 374	26.04	2568	26.15	0.11	0.003
Missing	30 948	5.36	789	8.03	2.67	0.107
Cerebrovascular disease						
No	519 703	90	8557	87.14	−2.86	−0.09
Yes	31 971	5.54	682	6.95	1.41	0.058
Unknown	25 785	4.47	581	5.92	1.45	0.065

BMI, body mass index.

After inverse probability weighting, most baseline covariates were balanced across cancer status, apart from white (SMD=0.119) and Asian (SMD=−0.103) ethnicities in the first year post-MI, while in the second year all baseline covariates were balanced (table 2 and online supplemental table 4).

**Table 2:** Weighted baseline characteristics of the study population after inverse probability weighting in the first year after myocardial infarction

Characteristics	Non-cancer/comparison cohort N (%)/mean (SD)		Cancer cohort N (%)/mean (SD)		Difference in proportions/means between groups	Standardised mean difference
Total	577 459	100	9820	100	–	–
Age	73.18	12.5	73.13	10.3	−0.05	−0.004
Gender						
Male	400 274.60	69.32	6813	69.38	0.06	0.001
Female	177 184.40	30.68	3007	30.62	−0.06	−0.001
Ethnicity						
White	511 723.59	88.62	9045	92.11	3.49	0.119
Asian	28 015.78	4.85	281	2.86	−1.99	−0.103
Black	4843.15	0.84	87	0.89	0.05	0.005
Other	21 378.67	3.7	277	2.82	−0.88	−0.05
Not known	11 497.82	1.99	130	1.32	−0.67	−0.052
Geographical region						
East Midlands	41 072.84	7.11	637	6.49	−0.62	−0.025
East of England	63 702.05	11.03	1165	11.86	0.83	0.026
London	71 714.20	12.42	1148	11.69	−0.73	−0.022
North East	39 262.39	6.8	614	6.25	−0.55	−0.022
North West	85 385.73	14.79	1558	15.87	1.08	0.03
South East	80 078.40	13.87	1369	13.94	0.07	0.002
South West	68 691.89	11.9	1250	12.73	0.83	0.025
West Midlands	63 348.70	10.97	947	9.64	−1.33	−0.044
Yorkshire and the Humber	64 202.81	11.12	1132	11.53	0.41	0.013
BMI category						
Underweight	8054.43	1.39	217	2.21	0.82	0.061
Normal	108 793.79	18.84	1980	20.16	1.32	0.033
Overweight	128 816.23	22.31	1929	19.64	−2.67	−0.065
Obese	71 958.71	12.46	929	9.46	-3	−0.096
Very obese	6702.92	1.16	81	0.82	−0.34	−0.034
Missing	253 132.91	43.84	4684	47.7	3.86	0.078
Hypertension						
No	270 155.02	46.78	4598	46.82	0.04	0.001
Yes	279 875.15	48.47	4757	48.44	−0.03	−0.000
Unknown	27 428.83	4.75	465	4.74	−0.01	−0.001
Hypercholesterolaemia						
No	303 756.73	52.6	5164	52.59	−0.01	−0.000
Yes	244 872.76	42.41	4167	42.43	0.02	0.001
Unknown	28 829.51	4.99	489	4.98	−0.01	−0.001
Diabetes						
No	431 219.81	74.68	7336	74.7	0.02	0.001
Yes	134 854.51	23.35	2291	23.33	−0.02	−0.001
Unknown	11 384.67	1.97	193	1.97	0	−0.000
Falls and fractures						
No	530 832.94	91.93	9030	91.96	0.03	0.001
Yes	46 626.06	8.07	790	8.04	−0.03	−0.001
Previous bleeding						
No	450 012.66	77.93	7661	78.01	0.08	0.002
Yes	127 446.34	22.07	2159	21.99	−0.08	−0.002
Renal disease						
No	447 410.74	77.48	7619	77.59	0.11	0.003
Yes	130 048.26	22.52	2201	22.41	−0.11	−0.003
Smoking						
People who smoke	165 426.54	28.65	2817	28.69	0.04	0.001
People who have never smoked	214 456.53	37.14	3646	37.13	−0.01	−0.000
People who have quit smoking	150 967.20	26.14	2568	26.15	0.01	0.000
Missing	46 608.74	8.07	789	8.03	−0.04	−0.001
Cerebrovascular disease						
No	501 473.25	86.84	8557	87.14	0.3	0.009
Yes	43 475.19	7.53	682	6.95	−0.58	−0.023
Unknown	32 510.56	5.63	581	5.92	0.29	0.012

BMI, body mass index.

The prevalence of each cancer type is depicted in online supplemental tables 5 and 6 in the first and second years post-MI, allowing that individuals could have more than one type of cancer. Prostate, colorectal, lung, bladder and breast cancers were the most common malignancies.

Among 587 279 patients with MI, 9820 (1.7%) were diagnosed with cancer in the 1-year preceding their MI while 577 459 (98.3%) were not. Stratifying by MI, there were 254 046 patients with STEMI, of whom 3494 had cancer. There were 333 233 patients with NSTEMI, of whom 6326 had cancer. Among patients with STEMI, 190 234 patients received coronary stenting as part of their management, of whom 2015 patients had cancer. A higher proportion of patients with STEMI without cancer underwent coronary stenting compared with patients with cancer with STEMI (75.1% vs 57.7%). 138 303 patients with NSTEMI underwent coronary stenting, of whom 1587 had cancer. Compared with patients with cancer, a higher proportion of non-cancer NSTEMI patients received coronary stenting (41.8% vs 25.1%). 56 125 patients died in the first year of whom 3755 had cancer. 24 874 patients were lost to follow-up.

In the second year after MI, 506 280 patients were identified, 5666 (1.1%) had cancer while 500 614 (98.9%) did not. Of 222 845 patients with STEMI, 2067 had cancer. Of 283 435 patients with NSTEMI, 3599 had cancer.

### Clinical outcomes

The rate for bleeding in the first year post-MI was higher in patients with cancer compared with patients without cancer (HR: 1.53, 95% CI 1.45 to 1.62, p<0.001) (figure 2). The rate for bleeding in patients with MI with cancer remained higher in the second year post-MI (HR: 1.42, 95% CI 1.29 to 1.56, p<0.001) (figure 3). Using flexible parametric modelling, we modelled the effect of cancer on bleeding as a time-dependent effect, demonstrating that the increased rate of bleeding in the cancer group did not appear to vary by time since MI diagnosis (figure 4).

**Figure 2:**
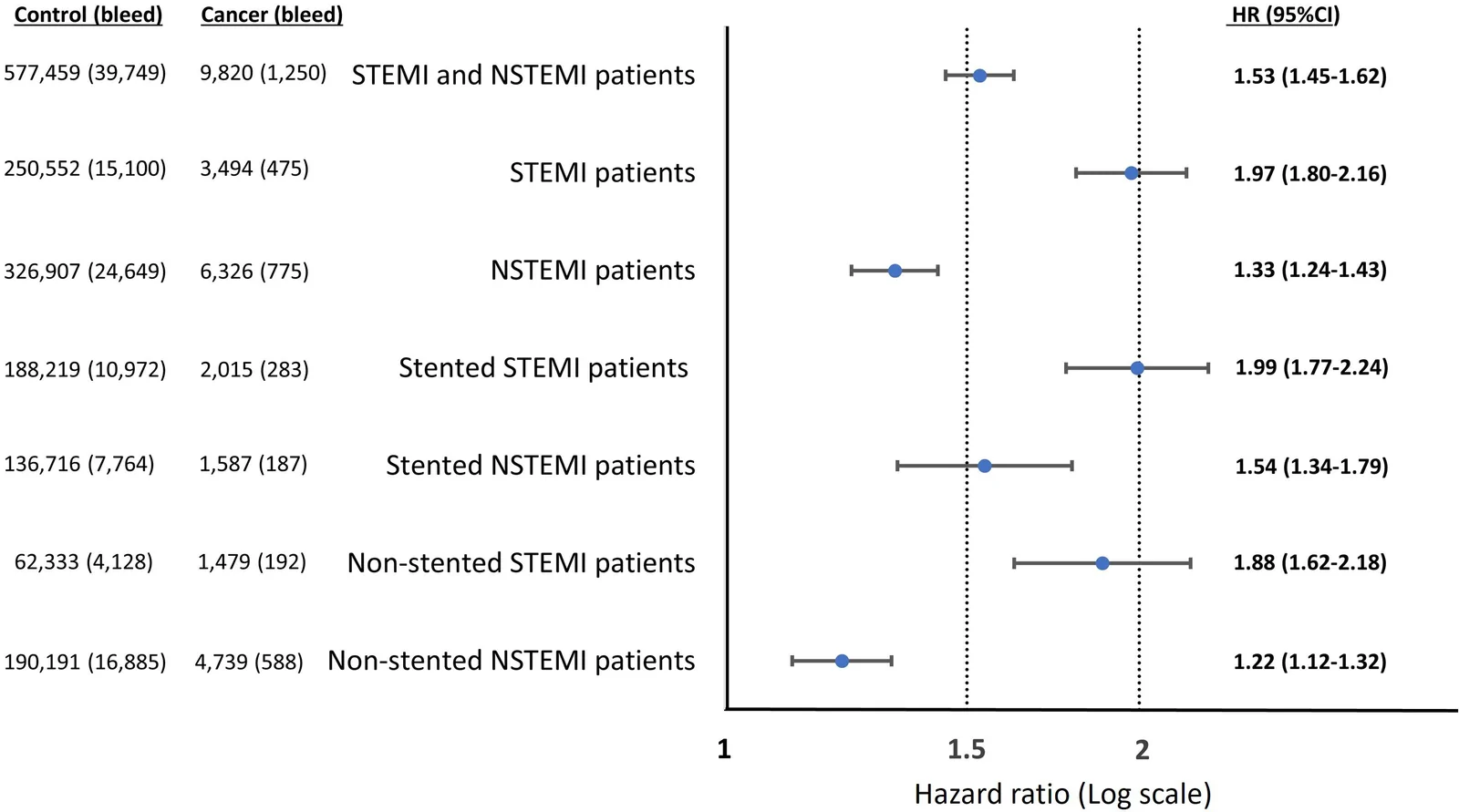
Forest plot demonstrating HRs and 95% CIs for presentation to hospital for bleeding comparing patients with cancer to patients without cancer in the first year after MI stratified by type of MI and coronary stent status (after inverse probability weighting, log scale). MI, myocardial infarction; NSTEMI, non-STEMI; STEMI, ST-elevation MI.

**Figure 3:**
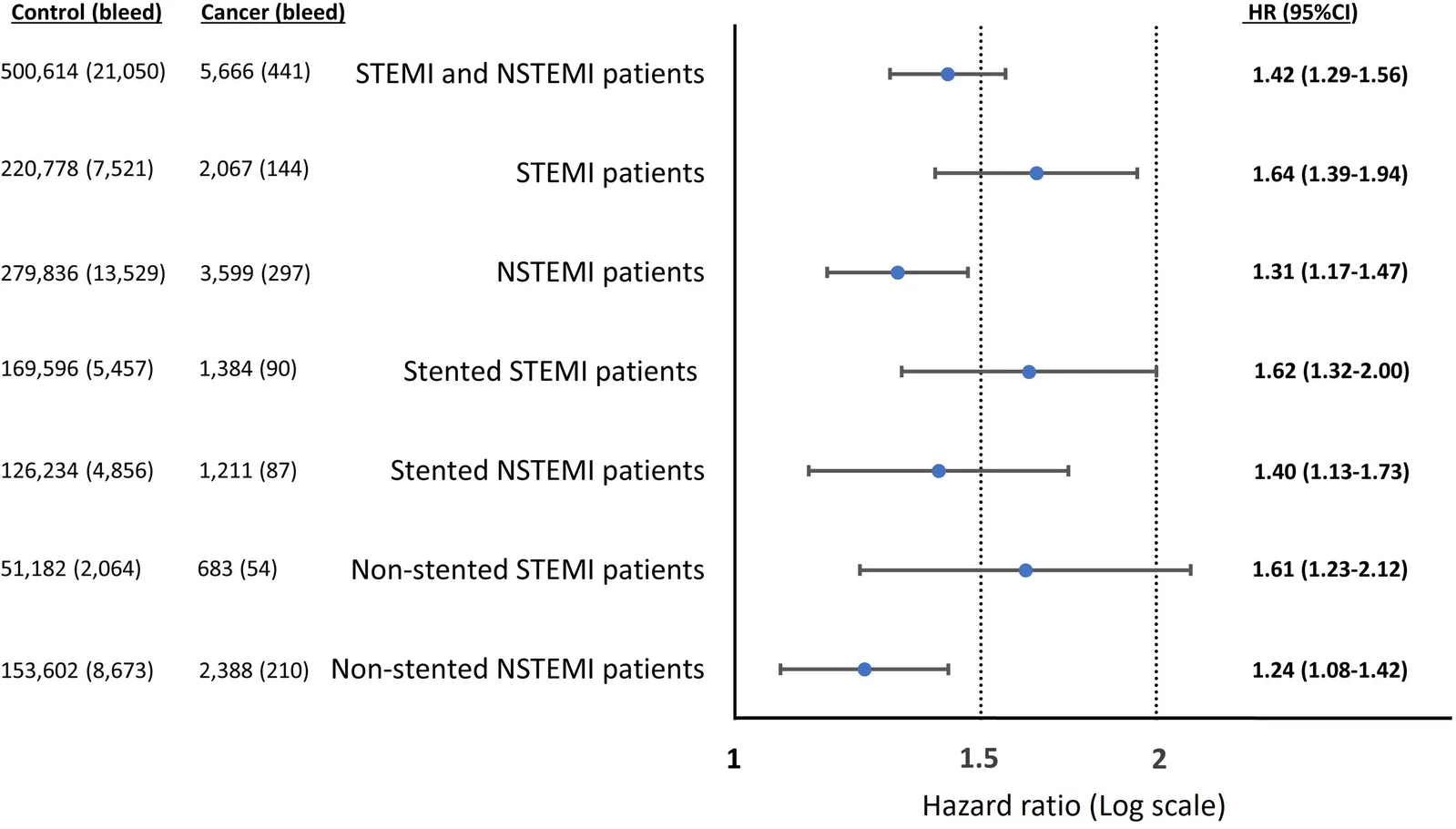
Forest plot demonstrating HRs and 95% CIs for presentation to hospital for bleeding comparing patients with cancer to patients without cancer in the second year after MI stratified by type of MI and coronary stent status (after inverse probability weighting, log scale). MI, myocardial infarction; NSTEMI, non-STEMI; STEMI, ST-elevation MI.

**Figure 4:**
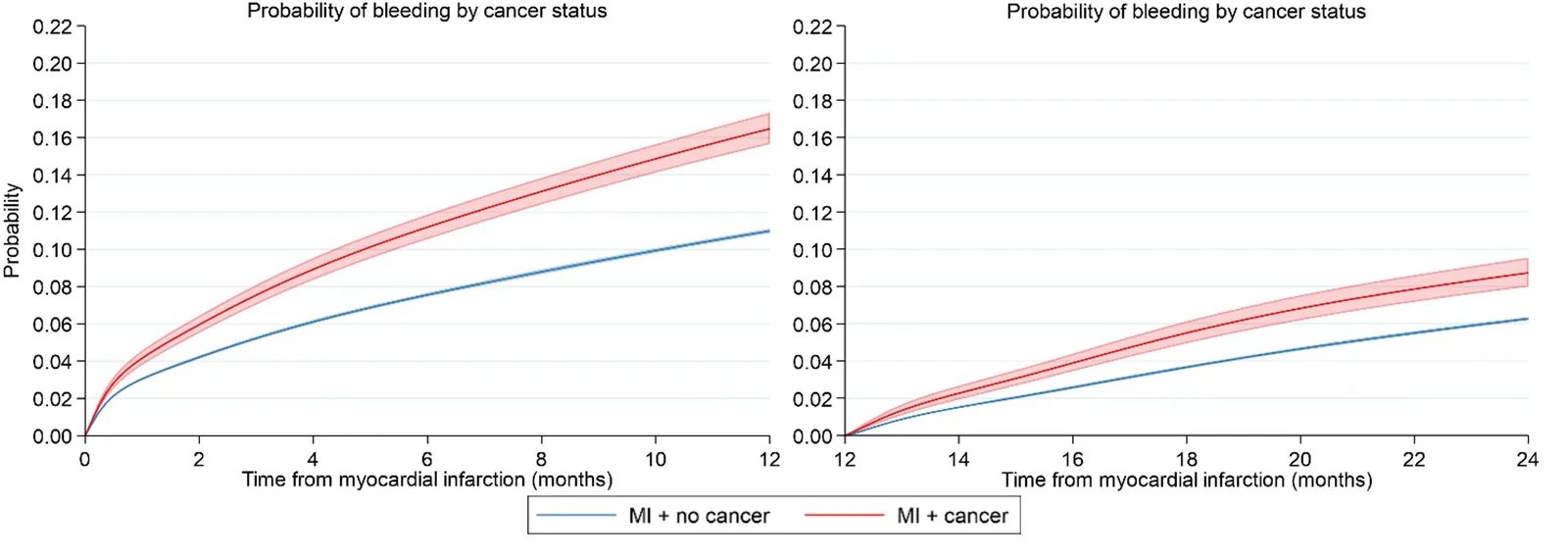
Probability of presentation to hospital for bleeding, comparing patients with cancer to patients without cancer in the first and second year after MI using flexible parametric methods. MI, myocardial infarction.

Stratifying by MI type and stent status in the first year after MI, patients with cancer remain at an increased bleeding risk (figure 2) (online supplemental figures 2–13). The rate of bleeding was greater in patients with cancer following STEMI compared with those with NSTEMI. The highest rate for bleeding was seen in patients with STEMI who had coronary stenting (HR: 1.99, 95% CI 1.77 to 2.24, p<0.001). Apart from breast cancer, the rate of bleeding was higher in all cancer types when compared with patients without cancer (figure 5) (online supplemental figures 14–23). The highest rate was observed in patients with bladder cancer (HR: 2.38, 95% CI 2.03 to 2.79, p<0.001).

**Figure 5:**
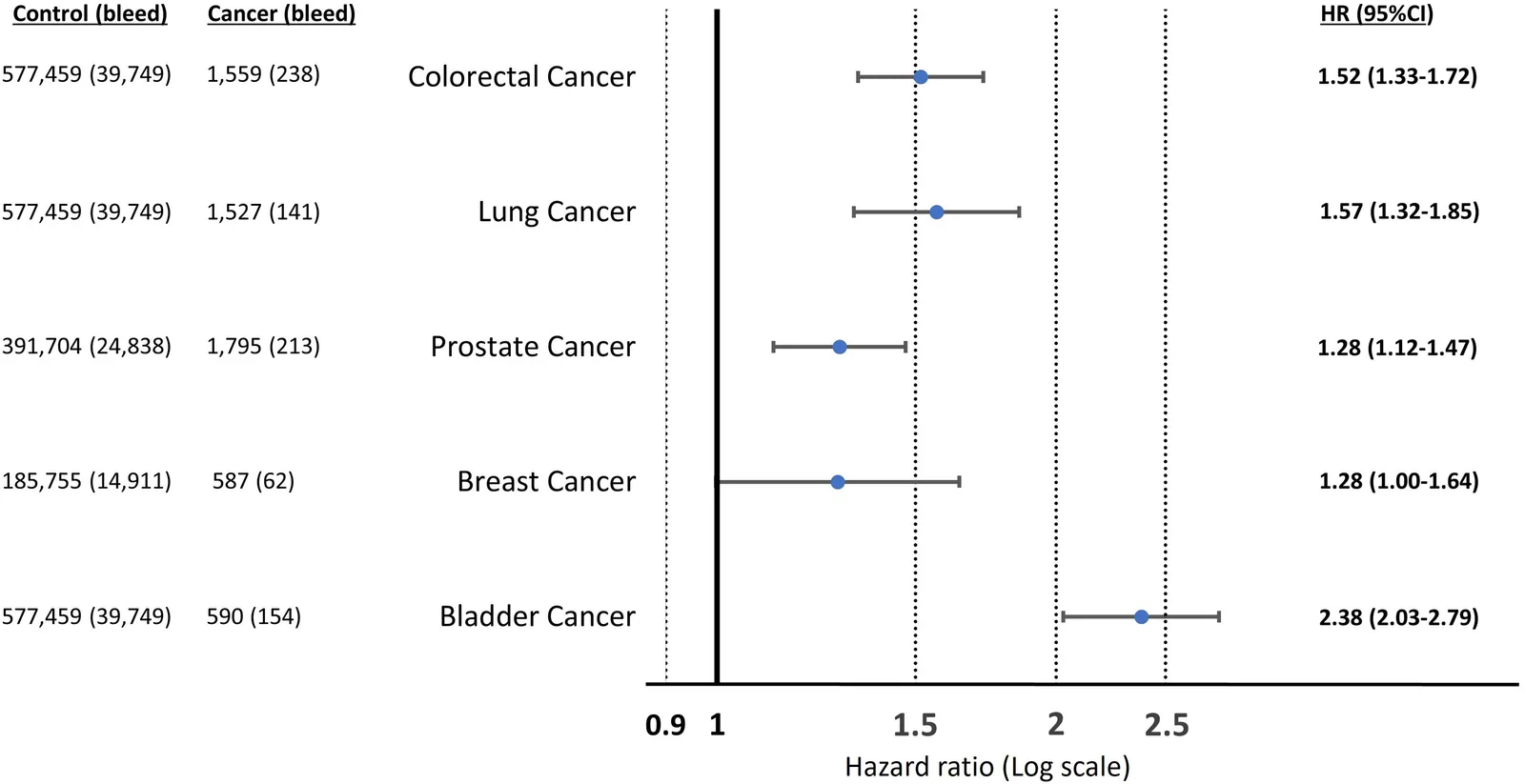
Forest plot demonstrating HRs and 95% CIs for presentation to hospital for bleeding comparing patients with cancer to patients without cancer in the first year after myocardial infarction stratified by cancer type (after inverse probability weighting, log scale).

Stratifying by MI type and stent status in the second year after MI, patients with cancer remained at an increased bleeding risk (figure 3) (online supplemental figures 2–13). The highest rate for bleeding in the second year post-MI was seen in patients with STEMI with cancer (HR: 1.64, 95% CI 1.39 to 1.94, p<0.001). Patients with lung, prostate and bladder cancer remained at a statistically significant increased risk of bleeding (figure 6) (online supplemental figures 14–23). The highest rate was still observed in patients with bladder cancer (HR: 1.92, 95% CI 1.42 to 2.60, p<0.001).

**Figure 6:**
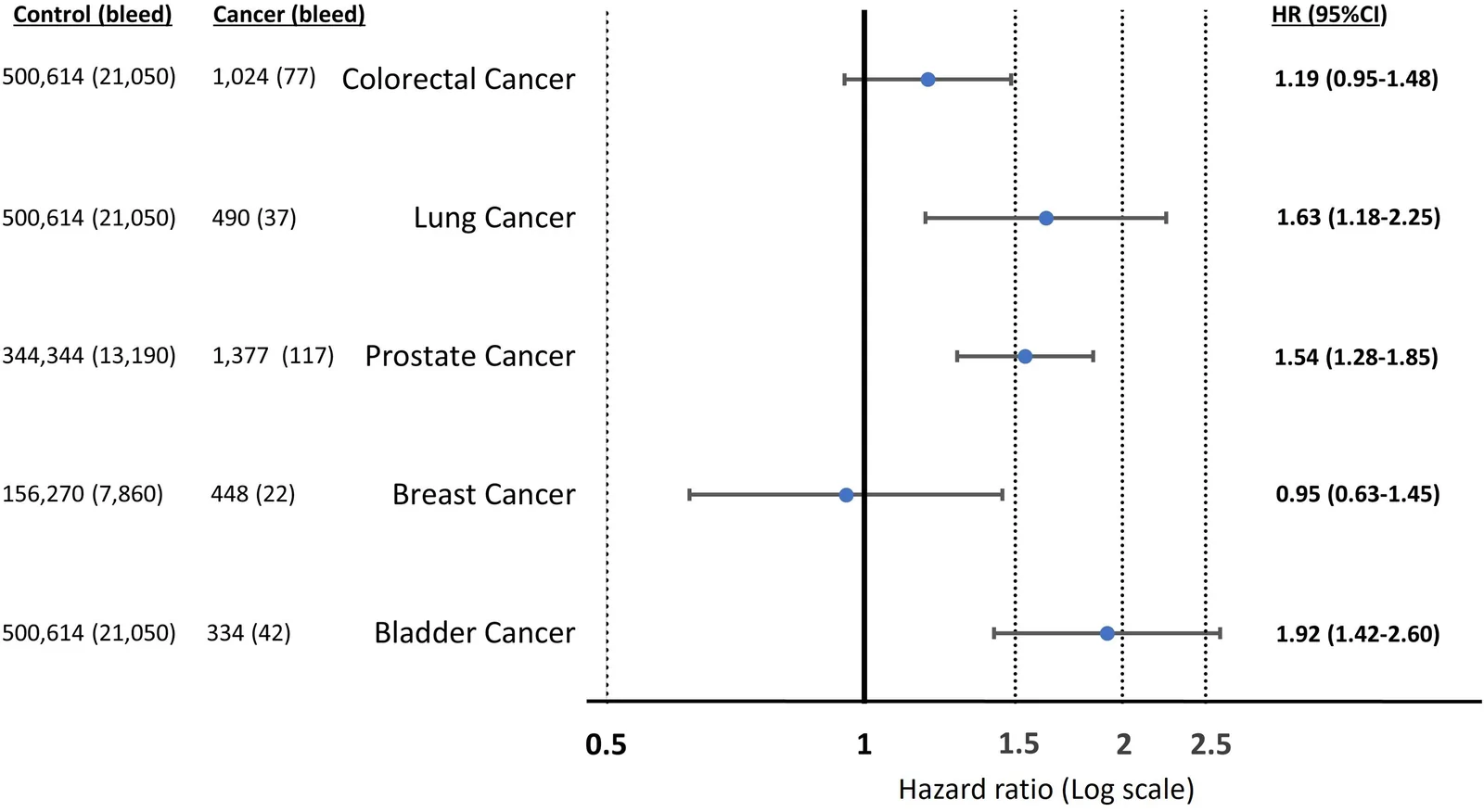
Forest plot demonstrating HRs and 95% CIs for presentation to hospital for bleeding comparing patients with cancer to patients without cancer in the second year after myocardial infarction stratified by cancer type (after inverse probability weighting, log scale).

Model fit was assessed with the Cox model. The assumptions of the Cox model were assessed and did not violate the proportional hazards assumption. Sensitivity analysis that excluded patients who had a bleeding event prior to the study period confirmed that patients with cancer have a higher bleeding risk (online supplemental figures 24–51). The higher rates of bleeding among the cancer group compared with the non-cancer group persisted across all the groups when stratified by types of MI and cancer, apart from colorectal and breast cancer in the second year post-MI. On excluding iron deficiency anaemia from the definition of bleeding, the effect size remained similar (online supplemental figures 52 and 53). Bootstrapped CIs were similar to those estimated from the Cox model (online supplemental figure 54).

## Discussion

We present data on a large population from England, investigating the risk of hospitalisation for bleeding in patients with MI, with and without cancer. We aimed to demonstrate the risk compounded by a recent diagnosis of cancer, type of MI and the stenting strategy adopted. The distribution of cancer types in patients with MI was consistent with previous studies.^13 41^ Patients with cancer were found to be older compared with patients without cancer.^42 43^

We observed a lower use of PCI in patients with cancer, as seen previously.^13 42^ This is expected as the time frame of our study predates the European Society of Cardiology (ESC) cardio-oncology guidelines^19^ and studies suggesting patients with cancer should not be excluded from receiving invasive management strategies.^13 44^ There remains no specific randomised trial evaluating the benefits and risks of invasive management in patients with cancer, and these data do not determine the appropriateness or otherwise of clinical decision-making on the optimal interventional versus non-interventional strategy in this study population.

Patients with cancer were at an increased bleeding rate, consistent with prior literature.^13 45^ The bleeding rate in patients with STEMI and cancer was higher compared with patients with NSTEMI and cancer in the first year. This could be explained by differences in prescribing, where patients with STEMI are expected to be treated more aggressively with DAPT and adhere to a stricter antiplatelet regimen. When stratified by MI type and stent status, patients with cancer remained at an elevated risk of bleeding even after 1 year, by which time patients would likely have had their second antiplatelet discontinued. While this is not unexpected, we anticipated a larger reduction in bleeding risk on the cessation of the second antiplatelet.

We observed that different cancer types conferred a different bleeding risk. In the first year, patients with colorectal, lung, prostate and bladder cancer were at an increased bleeding risk. Patients with bladder cancer were at the highest risk of bleeding, a finding not seen in previous large population studies.^13^ Patients with breast cancer were not at an increased risk of bleeding. Patients with colorectal cancer did not appear to be at a significant bleeding risk in the second year post-MI. The dissipation of bleeding risk seen in the colorectal cancer group in the second year after MI potentially suggests that bleeding risk in this cohort is particularly mediated by the addition of a second antiplatelet, as opposed to lung, prostate and bladder cancer, where their bleeding risk may be mainly cancer-mediated. A recent study observed higher rates of bleeding in patients with cancer, which occurred mainly after DAPT discontinuation despite a short DAPT regime, further suggesting this may be predominantly cancer-mediated.^46^

Characterising bleeding risks based on different malignancies may allow for a more personalised assessment of risk, potentially enabling future optimisation of antiplatelet strategies in patients with cancer, including a shorter duration of antiplatelet use. There remains a paucity of data on performing a stratified analysis by cancer type within an MI population due to sample size limitations. Importantly, cancer is associated with a higher mortality after bleeding events, highlighting the importance of accurate assessment of bleeding risk in this cohort.^46^

The number of patients with cancer receiving PCI is likely to increase in the future as growing evidence and guidelines recommend PCI in patients with cancer.^13 19 43^ Balancing ischaemic and bleeding risk is challenging in patients with cancer, as contemporary risk scores used are not validated in patients with cancer. Further prospective evaluation of treatment variation based on cancer-specific risk scores has the potential to improve outcomes in patients with cancer presenting with MI.

Our study focused on the highest risk period for bleeding in patients with cancer (within 1 year of diagnosis) and severe bleeding events. Previous literature suggests that patients with PCI and a recent cancer diagnosis, defined as a diagnosis ≤1 year, were at the highest risk of bleeding.^47^ Linkage with HES ensured a focus on bleeding events that were severe enough to require hospital presentation. We additionally focused on MI type, on differences between those who did and did not receive PCI and measured outcomes within and after the first year. VICORI does not currently include prescribed medication data, but the current analyses provide insights into the temporal dynamics of bleeding risk including an earlier period with likely use of DAPT and a latter period with likely use of SAPT, both characterised by elevated risk of bleeding. Excluding patients with an indication for anticoagulation or concomitant warfarin use was essential to isolate the bleeding risk specifically attributable to antiplatelet use post-MI. Inclusion of these patients would introduce significant confounding. The effect of anticoagulation in this population would be an area of future study.

Our study benefits from the large sample size resulting from data-linkage of national datasets, representative of a real-world population. Data linkage allowed us to capture data on the time of cancer diagnosis, allowing for refined characterisation of the included patient population. Our large sample size allowed us to stratify by MI type, coronary stenting and cancer type. The analytical methodology adopted in this study allowed us to gain insights on causal estimates to help us understand the impact of cancer on bleeding risk post-MI. Current guidelines do not distinguish between different malignancies, likely due to lack of data, limited by small sample sizes of current studies.^19^

### Limitations

VICORI data is not linked to prescribing data, precluding direct investigation of treatment effects of prescribed antiplatelets. Antiplatelet usage was not reliably recorded in the audits used. We did not capture the use of cancer therapy, surveillance interventions, in relation to the MI, which could be an important risk factor for bleeding. As with most real-world data studies, we were subject to missing data; this could have introduced systematic bias if there were differences in ‘missingness’ between people with and without cancer. We also recognise that metastatic disease may be associated with an increase in bleeding events.^13^ We deliberately excluded patients who have an indication for anticoagulation to focus on a non-anticoagulated population, to assess the bleeding risk in patients related to standard guideline-based post-MI antiplatelets. We accept that a population requiring anticoagulation post-MI merits further study. We also did not exclude patients who had a previous history of cancer outside of the 1-year pre-MI period, nor patients with a new cancer diagnosis after their MI. These two patient groups would ultimately be included in the control population with no cancer. Cancer diagnosed prior to the study period was not extracted as part of this linked database, and so we cannot directly estimate the size of this group consistently for all those included in the study. However, we anticipate that the number of these patients is likely to be small compared with the overall control population and the impact on the data presented would, if anything, be to reduce the differences between the studies groups.

## Conclusions

Patients with cancer are at increased risk of bleeding, requiring hospital presentation after MI compared with patients without cancer. This risk differed by type of MI, stent status and cancer type. Among the cancer types, patients with bladder cancer have the highest risk of bleeding. Patients with cancer have a persistent, increased bleeding risk beyond the 1-year post-MI period, although lower compared with the first year post-MI. Further work is needed to identify optimal treatment strategies, which favourably balance the ischaemic and bleeding risk in this population.

## Supplementary material

10.1136/heartjnl-2025-326524 online supplemental file 1

## Data Availability

Data are available upon reasonable request.
