# Supplementary material for: Impact of a recent cancer diagnosis on bleeding risk after myocardial infarction
Source: Heart. 2025 Dec 30;112(21):e326524. doi: 10.1136/heartjnl-2025-326524 (PMC13691686; doi:10.1136/heartjnl-2025-326524)
Supplement: Supplementary file 1 [file heartjnl-112-21-s001.pdf]

The Impact of a Recent Cancer Diagnosis on Bleeding Risk after Myocardial Infarction

Supplemental Material

Kok Weng Ow MRCP,[1,2] Freya Tyrer PhD,[2-4] Freek Van Den Berg MD,[5] Jennifer Lai PhD,[2] Sally Vernon MMath,[2] Lizz Paley MSc,[2] Florian A. Wenzl MD,[1,2,6,7] Clive Weston MRCP,[8] Mark J. Rutherford PhD,[3] Paul C. Lambert PhD,[9, 10] Mark de Belder MD,[11, 12] John Deanfield FMedSci,[13, 14] Michael D. Peake FRCP,[15] and David Adlam DPhil,[1, 16]

Affiliations

1. Department of Cardiovascular Sciences, University of Leicester and NIHR Leicester Biomedical Research Centre, Leicester, United Kingdom of Great Britain & Northern Ireland
2. National Disease Registration Service, NHS England, London, United Kingdom of Great Britain & Northern Ireland
3. Department of Population Health Sciences, University of Leicester, Leicester, United Kingdom of Great Britain & Northern Ireland
4. Observational & Pragmatic Research Institute, United Kingdom of Great Britain & Northern Ireland
5. Department of Experimental Cardiology, University Medical Centre Groningen, Groningen, Netherlands
6. Center for Molecular Cardiology, University of Zurich, Wagistrasse 12, 8952 Schlieren, Switzerland
7. Department of Clinical Sciences, Karolinska Institutet, Stockholm, Sweden.
8. Glangwili General Hospital, Carmarthen, Wales, United Kingdom of Great Britain & Northern Ireland
9. Cancer Registry of Norway, Norwegian Institute of Public Health, Oslo, Norway
10. Department of Medical Epidemiology and Biostatistics, Karolinska Institutet, Stockholm, Sweden
11. National Institute for Cardiovascular Outcomes Research (NICOR), NHS Arden and Greater East Midlands Commissioning Support Unit, Leicester, United Kingdom of Great Britain & Northern Ireland
12. William Harvey Research Institute, Queen Mary University London, United Kingdom of Great Britain & Northern Ireland
13. Institute of Cardiovascular Science, University College London, London, United Kingdom of Great Britain & Northern Ireland
14. National Institute of Cardiovascular Outcomes Research (NICOR), United Kingdom of Great Britain & Northern Ireland
15. University of Leicester, Leicester, United Kingdom of Great Britain & Northern Ireland
16. Leicester British Heart Foundation Centre of Research Excellence, United Kingdom of Great Britain & Northern Ireland

Correspondence

Professor David Adlam, Department of Cardiovascular Sciences, University of Leicester, Leicester, United Kingdom; da134@leicester.ac.uk

Supplemental Table 1: ICD-10 Cancer diagnoses and groupings

ICD-10 code(s)	Cancer site group
C15	Oesophagus
C16	Stomach
C17	Small intestine
C18 to C20	Colorectal
C21	Anus
C22	Liver
C25	Pancreas
C32	Larynx
C33 and C34	Lung
C43	Melanoma
C45	Mesothelioma
C50	Breast
C51	Vulva
C53	Cervix
C54 and C55	Uterus
C56 to C57.7	Ovary
C61	Prostate
C62	Testis
C64	Kidney
C67[a]	Bladder
C71	Brain
C73	Thyroid
C81	Hodgkin lymphoma
C82 to C86	Non-Hodgkin lymphoma
C90	Myeloma
C91 to C95	Leukaemia

Based on (Battisti, Welch et al. 2022). In line with this work, only cancers defined as 'malignant' (primary or secondary/metastatic) will be included. ICD-10 code C44 (non-melanoma cancers) will not be included. [1]

[a] Patients with bladder cancer were included in our analysis if their bladder cancer was defined as invasive according to ICD O2. This means that the risk of bleeding in patients with very early-stage bladder cancer, (Ta or Tis, where the carcinoma has not invaded the subepithelial connective tissue) has not been assessed. These patients make up approximately half of all diagnoses of 'bladder cancer.

Supplemental Table 2: ICD-10 codes for major bleeding & iron deficiency anaemia

**Major Bleeding**		
**Body area**	**ICD-10**	**Description**
Intracranial	I60.x	Subarachnoid haemorrhage
	I61.x	Intracerebral haemorrhage
	I62.0	Subdural haemorrhage (acute)(nontraumatic)
	I62.1	Nontraumatic extradural haemorrhage
	I62.9	Intracranial haemorrhage (nontraumatic), unspecified
Upper gastrointestinal	K92.0	Haematemesis
	K92.1	Melaena
	I85.0	Oesophageal varices with bleeding
	I98.20	Oesophageal varices in diseases classified elsewhere with bleeding
	I98.3	Oesophageal varices with bleeding in diseases classified elsewhere
	K22.10	Ulcer of oesophagus, acute with bleeding
	K22.12	Ulcer of oesophagus, acute with both bleeding and perforation
	K22.14	Ulcer of oesophagus, chronic or unspecified with bleeding
	K22.16	Ulcer of oesophagus, chronic or unspecified with both bleeding and perforation
	K25.0	Gastric ulcer, acute with haemorrhage
	K25.2	Gastric ulcer, acute with both haemorrhage and perforation
	K25.4	Gastric ulcer, chronic or unspecified with haemorrhage
	K25.6	Gastric ulcer, chronic or unspecified with both haemorrhage and perforation
	K26.0	Duodenal ulcer, acute with haemorrhage
	K26.2	Duodenal ulcer, acute with both haemorrhage and perforation
	K26.4	Duodenal ulcer, chronic or unspecified with haemorrhage
	K26.6	Duodenal ulcer, chronic or unspecified with both haemorrhage and perforation
	K27.0	Peptic ulcer, acute with haemorrhage
	K27.2	Peptic ulcer, acute with both haemorrhage and perforation
	K27.4	Peptic ulcer, chronic or unspecified with haemorrhage
	K27.6	Peptic ulcer, chronic or unspecified with both haemorrhage and perforation
	K28.0	Gastrojejunal ulcer, acute with haemorrhage
	K28.2	Gastrojejunal ulcer, acute with both haemorrhage and perforation
	K28.4	Gastrojejunal ulcer, chronic or unspecified with haemorrhage

	K28.6	Gastrojejunal ulcer, chronic or unspecified with both haemorrhage and perforation
	K29.0	Acute haemorrhagic gastritis
	K31.80	Angiodysplasia of stomach and duodenum with bleeding.
Lower gastrointestinal	K55.20	Angiodysplasia of colon with bleeding.
	K62.5	Haemorrhage of anus and rectum
	K92.2	Gastrointestinal haemorrhage, unspecified
Other bleeding	N02.0	Recurrent and persistent haematuria, minor glomerular abnormality
	N02.1	Recurrent and persistent haematuria, focal and segmental glomerular lesions
	N02.2	Recurrent and persistent haematuria, diffuse membranous glomerulonephritis
	N02.3	Recurrent and persistent haematuria, diffuse mesangial proliferative glomerulonephritis
	N02.4	Recurrent and persistent haematuria, diffuse endocapillary proliferative glomerulonephritis
	N02.5	Recurrent and persistent haematuria, diffuse mesangiocapillary glomerulonephritis
	N02.6	Recurrent and persistent haematuria, dense deposit disease
	N02.7	Recurrent and persistent haematuria, diffuse crescentic glomerulonephritis
	N02.8	Recurrent and persistent haematuria, other
	N02.9	Recurrent and persistent haematuria, unspecified
	K66.1	Haemoperitoneum
	N93.8	Other specified abnormal uterine and vaginal bleeding
	N93.9	Abnormal uterine and vaginal bleeding, unspecified
	N95.0	Postmenopausal bleeding
	R04.1	Haemorrhage from throat
	R04.2	Haemoptysis
	R04.8	Haemorrhage from other sites in respiratory passages
	R04.9	Haemorrhage from respiratory passages, unspecified
	R31.0	Gross hematuria.
	R31.1	Microscopic hematuria
	R31.8	Other and unspecified hematuria
	R58	Haemorrhage, not elsewhere classified
	D68.3	Haemorrhagic disorder due to circulating anticoagulants
	H35.6	Retinal haemorrhage

	H43.1	Vitreous haemorrhage
	H45.0	Vitreous haemorrhage in diseases classified elsewhere
	M25.0	Haemarthrosis
Additional codes	D50.0	Iron deficiency anaemia secondary to blood loss (chronic)
	D50.8	Other iron deficiency anaemia
	D50.9	Iron deficiency anaemia, unspecified

Based on Jun et al. [2]

Supplemental Figure 1: Directed Acyclic Graph

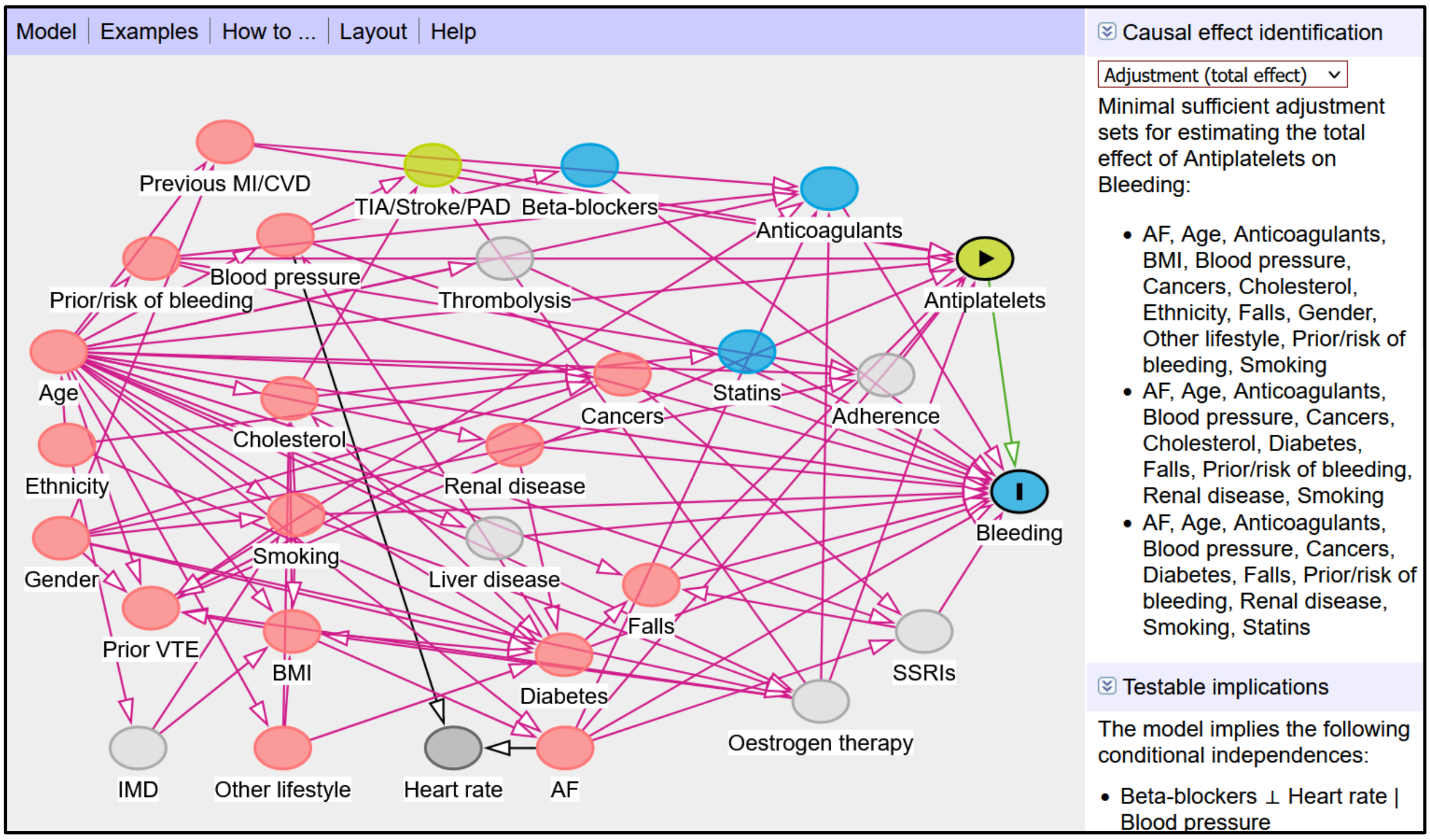

Supplemental Table 3: Unweighted Baseline Characteristics of the Study Population in the Second Year after Myocardial Infarction

Characteristics		Non-cancer/comparison cohort N(%)/ mean (SD)		Cancer cohort N(%)/ mean (SD)		Difference in proportions/ means between groups	Standardised mean difference
Total		500,614	100%	5,666	100%	-	-
Age		65.96	12.7	71.77	10.2	5.81	0.504
Gender	Male	344,344	68.78	4,041	71.32	2.54	0.055
	Female	156,270	31.22	1,625	28.68	-2.54	-0.055
Ethnicity	White	438,002	87.49	5,192	91.63	4.14	0.136
	Asian	29,124	5.82	180	3.18	-2.64	-0.128
	Black	4,609	0.92	55	0.97	0.05	0.005
	Other	21,694	4.33	192	3.39	-0.94	-0.049
	Not Known	7,185	1.44	47	0.83	-0.61	-0.057
Geographical Region	East Midlands	35,907	7.17	376	6.64	-0.53	-0.021
	East of England	51,905	10.37	686	12.11	1.74	0.055
	London	66,530	13.29	683	12.05	-1.24	-0.037
	North East	36,740	7.34	363	6.41	-0.93	-0.037
	North West	71,203	14.22	853	15.05	0.83	0.024
	South East	68,272	13.64	771	13.61	-0.03	-0.001
	South West	54,641	10.91	735	12.97	2.06	0.063
	West Midlands	59,938	11.97	587	10.36	-1.61	-0.051
	Yorkshire & Humber	55,478	11.08	612	10.8	-0.28	-0.009
BMI Category	Underweight	4,830	0.96	76	1.34	0.38	0.035
	Normal	89,907	17.96	1,122	19.8	1.84	0.047
	Overweight	126,071	25.18	1,321	23.31	-1.87	-0.044
	Obese	80,028	15.99	665	11.74	-4.25	-0.123
	Very Obese	8,414	1.68	57	1.01	-0.67	-0.059
	Missing	191,364	38.23	2,425	42.8	4.57	0.093
Hypertension	No	252,361	50.41	2,619	46.22	-4.19	-0.084
	Yes	230,918	46.13	2,809	49.58	3.45	0.069
	Unknown	17,335	3.46	238	4.2	0.74	0.038
Hypercholesterola-emia	No	251,399	50.22	2,831	49.96	-0.26	-0.005
	Yes	232,144	46.37	2,586	45.64	-0.73	-0.015
	Unknown	17,071	3.41	249	4.39	0.98	0.051
Diabetes	No	390,766	78.06	4,311	76.09	-1.97	-0.047
	Yes	103,167	20.61	1,244	21.96	1.35	0.033
	Unknown	6,681	1.33	111	1.96	0.63	0.049
Falls & Fractures	No	471,284	94.14	5,307	93.66	-0.48	-0.02
	Yes	29,330	5.86	359	6.34	0.48	0.02
Previous bleeding	No	456,385	91.17	4,511	79.62	-11.55	-0.331
	Yes	44,229	8.83	1,155	20.38	11.55	0.331
Renal disease	No	452,809	90.45	4,583	80.89	-9.56	-0.276
	Yes	47,805	9.55	1,083	19.11	9.56	0.276
Smoking	Current smoker	180,868	36.13	1,603	28.29	-7.84	-0.168
	Non-smoker	162,669	32.49	2,096	36.99	4.5	0.095
	Ex smoker	133,684	26.7	1,589	28.04	1.34	0.03
	Missing	23,393	4.67	378	6.67	2	0.086
Cerebrovascular disease	No	455,663	91.02	5,046	89.06	-1.96	-0.066
	Yes	24,106	4.82	305	5.38	0.56	0.026
	Unknown	20,845	4.16	315	5.56	1.4	0.065

Supplemental Table 4: Weighted Baseline Characteristics of the Study Population after Inverse Probability Weighting in the Second Year after Myocardial Infarction

Characteristics		Non-cancer/comparison cohort N(%)/ mean (SD)		Cancer cohort N(%)/ mean (SD)		Difference in proportions/ means between groups	Standardised mean difference
Total		500,614	100%	5,666	100%	-	-
Age		71.80	12.3	71.77	10.2	-0.03	-0.003
Gender	Male	356,813.19	71.28	4,041	71.32	0.04	0.001
	Female	143,800.81	28.72	1,625	28.68	-0.04	-0.001
Ethnicity	White	444,717.08	88.83	5,192	91.63	2.8	0.094
	Asian	25,471.01	5.09	180	3.18	-1.91	-0.096
	Black	4,199.62	0.84	55	0.97	0.13	0.014
	Other	19,559.11	3.91	192	3.39	-0.52	-0.028
	Not Known	6,667.18	1.33	47	0.83	-0.5	-0.049
Geographical Region	East Midlands	35,886.94	7.17	376	6.64	-0.53	-0.021
	East of England	54,537.92	10.89	686	12.11	1.22	0.038
	London	63,378.75	12.66	683	12.05	-0.61	-0.018
	North East	35,282.09	7.05	363	6.41	-0.64	-0.026
	North West	70,774.54	14.14	853	15.05	0.91	0.026
	South East	69,499.31	13.88	771	13.61	-0.27	-0.008
	South West	59,089.30	11.8	735	12.97	1.17	0.035
	West Midlands	56,716.16	11.33	587	10.36	-0.97	-0.031
	Yorkshire & Humber	55,449.00	11.08	612	10.8	-0.28	-0.009
BMI Category	Underweight	5,574.72	1.11	76	1.34	0.23	0.021
	Normal	94,925.76	18.96	1,122	19.8	0.84	0.021
	Overweight	120,348.27	24.04	1,321	23.31	-0.73	-0.017
	Obese	68,179.59	13.62	665	11.74	-1.88	-0.057
	Very Obese	6,210.45	1.24	57	1.01	-0.23	-0.022
	Missing	205,375.21	41.02	2,425	42.8	1.78	0.036
Hypertension	No	231,260.41	46.2	2,619	46.22	0.02	0.001
	Yes	248,277.54	49.59	2,809	49.58	-0.01	-0.000
	Unknown	21,076.05	4.21	238	4.2	-0.01	-0.000
Hypercholesterola-emia	No	250,166.20	49.97	2,831	49.96	-0.01	-0.000
	Yes	228,400.45	45.62	2,586	45.64	0.02	0.000
	Unknown	22,047.35	4.4	249	4.39	-0.01	-0.000
Diabetes	No	380,821.54	76.07	4,311	76.09	0.02	0.000
	Yes	109,963.81	21.97	1,244	21.96	-0.01	-0.000
	Unknown	9,828.65	1.96	111	1.96	0	-0.000
Falls & Fractures	No	468,817.12	93.65	5,307	93.66	0.01	0.001
	Yes	31,796.88	6.35	359	6.34	-0.01	-0.001
Previous bleeding	No	398,323.07	79.57	4,511	79.62	0.05	0.001
	Yes	102,290.93	20.43	1,155	20.38	-0.05	-0.001
Renal disease	No	404,606.24	80.82	4,583	80.89	0.07	0.002
	Yes	96,007.77	19.18	1,083	19.11	-0.07	-0.002
Smoking	Current smoker	141,520.24	28.27	1,603	28.29	0.02	0.000
	Non-smoker	185,225.06	37	2,096	36.99	-0.01	-0.000
	Ex smoker	140,364.70	28.04	1,589	28.04	0	0.000
	Missing	33,504.00	6.69	378	6.67	-0.02	-0.001
Cerebrovascular disease	No	442,169.75	88.33	5,046	89.06	0.73	0.023
	Yes	32,862.59	6.56	305	5.38	-1.18	-0.05
	Unknown	25,581.66	5.11	315	5.56	0.45	0.02

Supplemental Table 5: Number of Cancers by Anatomical Location in the First Year After MI

Cancer	n	%
1. Anus	40	0.4
2. Bladder	590	5.9
3. Brain	52	0.5
4. Breast	609	6.1
5. Cervix	22	0.2
6. Colorectal	1559	15.6
7. Hodgkin lymphoma	42	0.4
8. Kidney	339	3.4
9. Larynx	94	0.9
10. Leukaemia	341	3.4
11. Liver	114	1.1
12. Lung	1527	15.3
13. Melanoma	251	2.5
14. Mesothelioma	76	0.8
15. Myeloma	272	2.7
16. Non-hodgkin lymphoma	407	4.1
17. Oesophagus	312	3.1
18. Ovarian	108	1.1
19. Pancreas	207	2.1
20. Prostate	1798	18.0
21. Small intestine	39	0.4
22. Stomach	272	2.7
23. Testis	20	0.2
24. Thyroid	25	0.3
25. Uterus	141	1.4
26. Vulva	37	0.4
27. Other	696	7.0
Total	9990	100

Supplemental Table 6: Number of Cancers by Anatomical Location in the Second Year After MI

Cancer	n	%
1. Anus	30	0.5
2. Bladder	334	5.8
3. Brain	6	0.1
4. Breast	463	8.0
5. Cervix	11	0.2
6. Colorectal	1024	17.8
7. Hodgkin lymphoma	28	0.5
8. Kidney	219	3.8
9. Larynx	55	1.0
10. Leukaemia	201	3.5
11. Liver	39	0.7
12. Lung	490	8.5
13. Melanoma	203	3.5
14. Mesothelioma	23	0.4
15. Myeloma	155	2.7
16. Non-hodgkin lymphoma	259	4.5
17. Oesophagus	110	1.9
18. Ovarian	50	0.9
19. Pancreas	28	0.5
20. Prostate	1379	24.0
21. Small intestine	25	0.4
22. Stomach	116	2.0
23. Testis	17	0.3
24. Thyroid	19	0.3
25. Uterus	91	1.6
26. Vulva	22	0.4
27. Other	360	6.3
Total	5757	100

Supplemental Figures for flexible parametric methods, comparing the first and second year after MI

Supplemental Figures 2 & 3 STEMI only

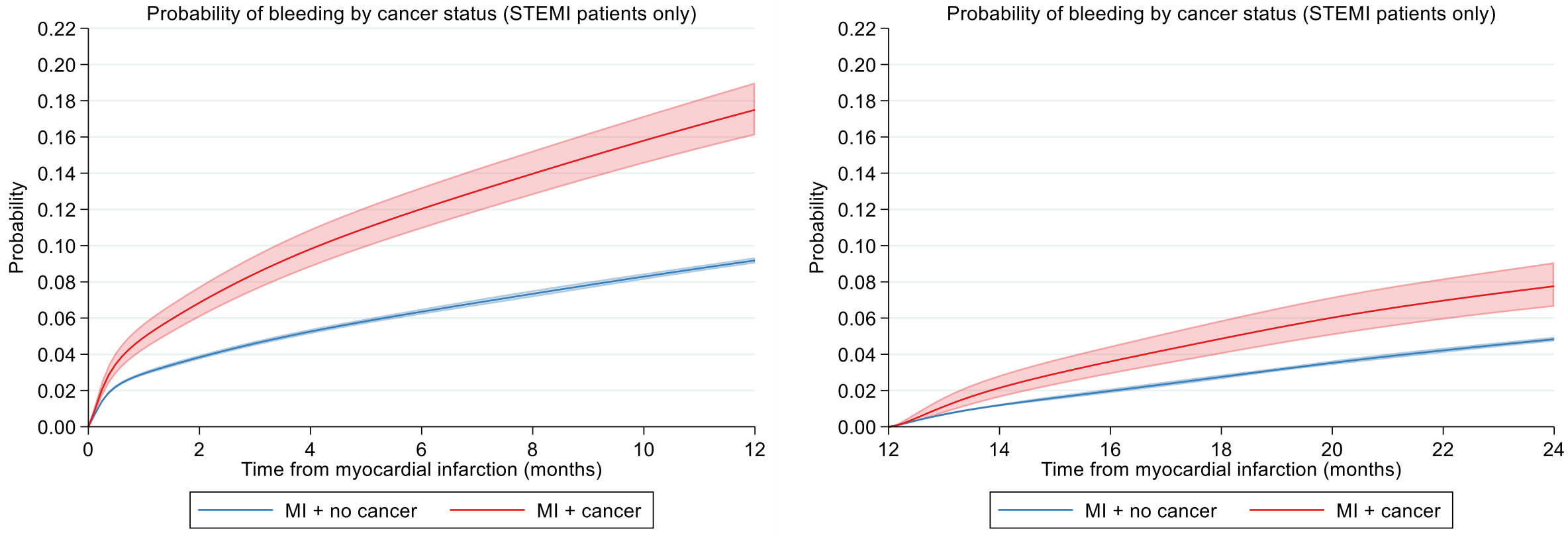

Probability of presentation to hospital for bleeding, comparing cancer patients to non-cancer patients in the first year (left) and second year (right) after MI using flexible parametric methods, after inverse probability weighting (STEMI patients only).

Supplemental Figures 4 & 5 NSTEMI Only

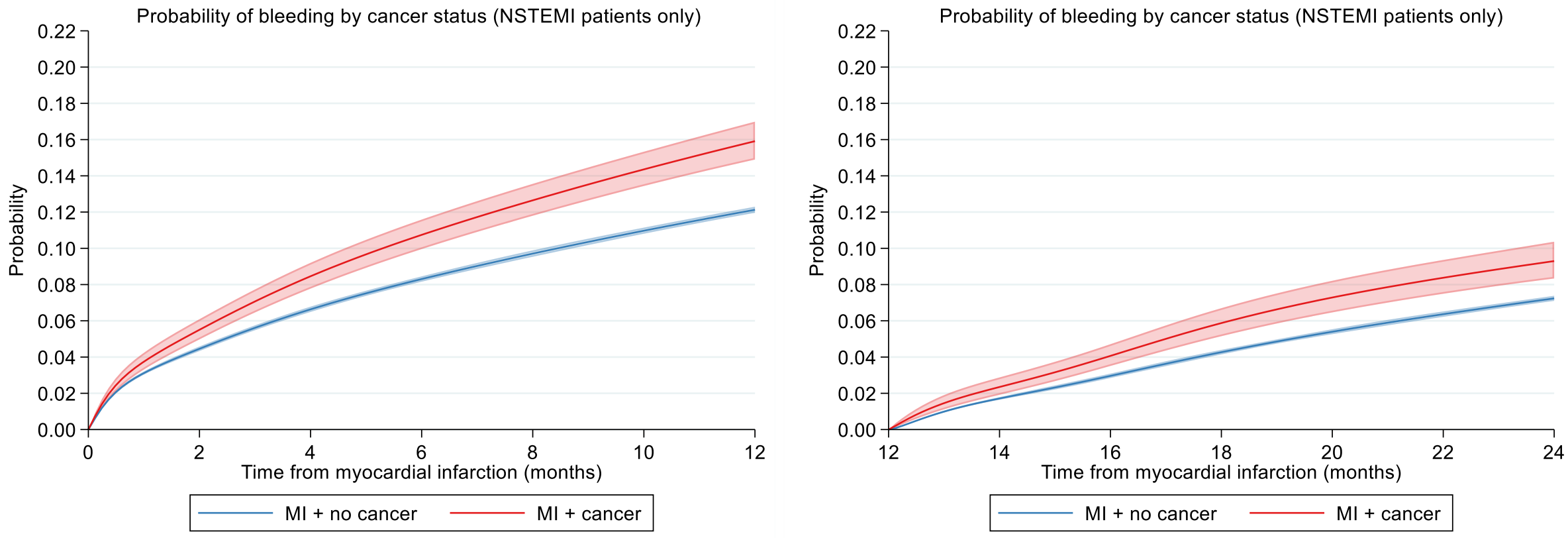

Probability of presentation to hospital for bleeding, comparing cancer patients to non-cancer patients in the first year (left) and second year (right) after myocardial infarction using flexible parametric methods, after inverse probability weighting (NSTEMI patients only).

Supplemental Figures 6 & 7 Stented STEMI only

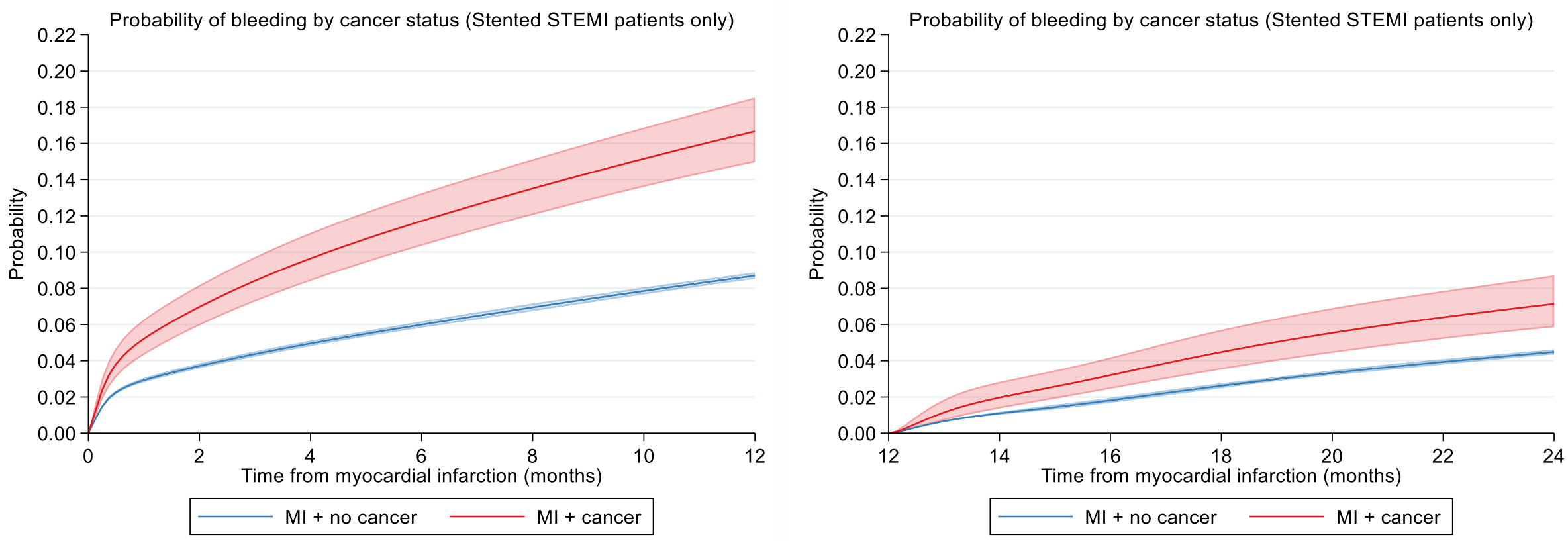

Probability of presentation to hospital for bleeding, comparing cancer patients to non-cancer patients in the first year (left) and second year (right) after myocardial infarction using flexible parametric methods, after inverse probability weighting (stented STEMI patients only).

Supplemental Figures 8 & 9 Stented NSTEMI

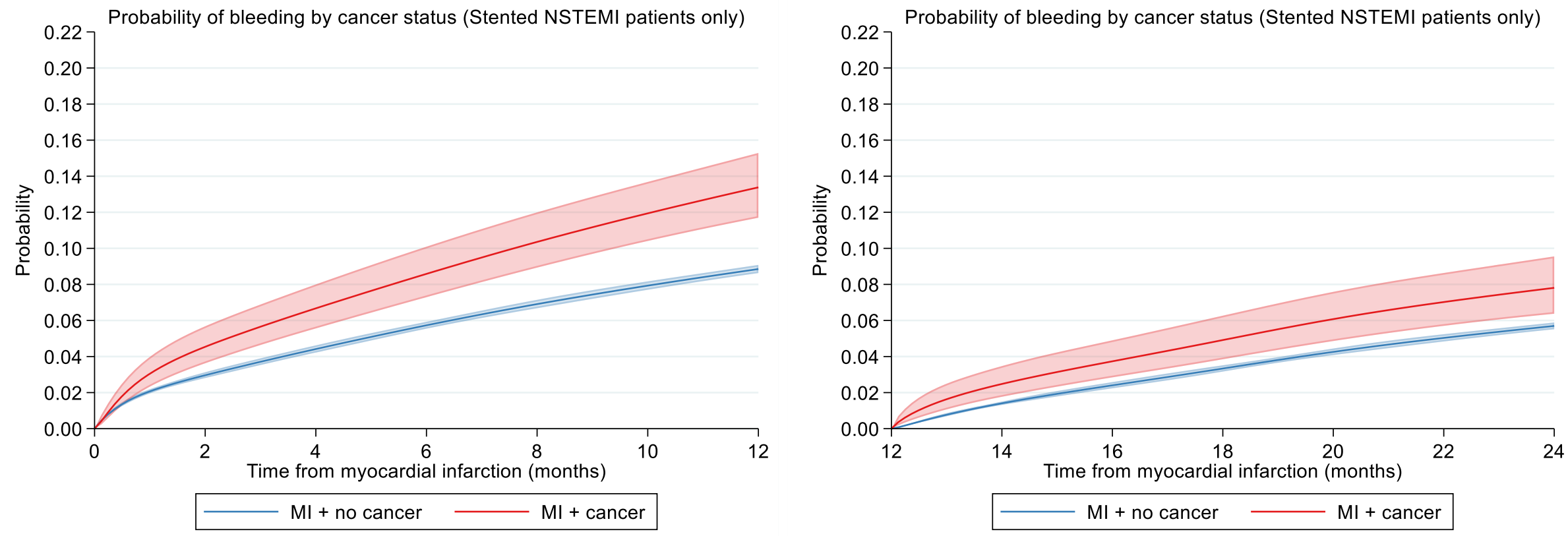

Probability of presentation to hospital for bleeding, comparing cancer patients to non-cancer patients in the first year (left) and second year (right) after myocardial infarction using flexible parametric methods, after inverse probability weighting (stented NSTEMI patients only).

Supplemental Figures 10 & 11 Non-stented STEMI

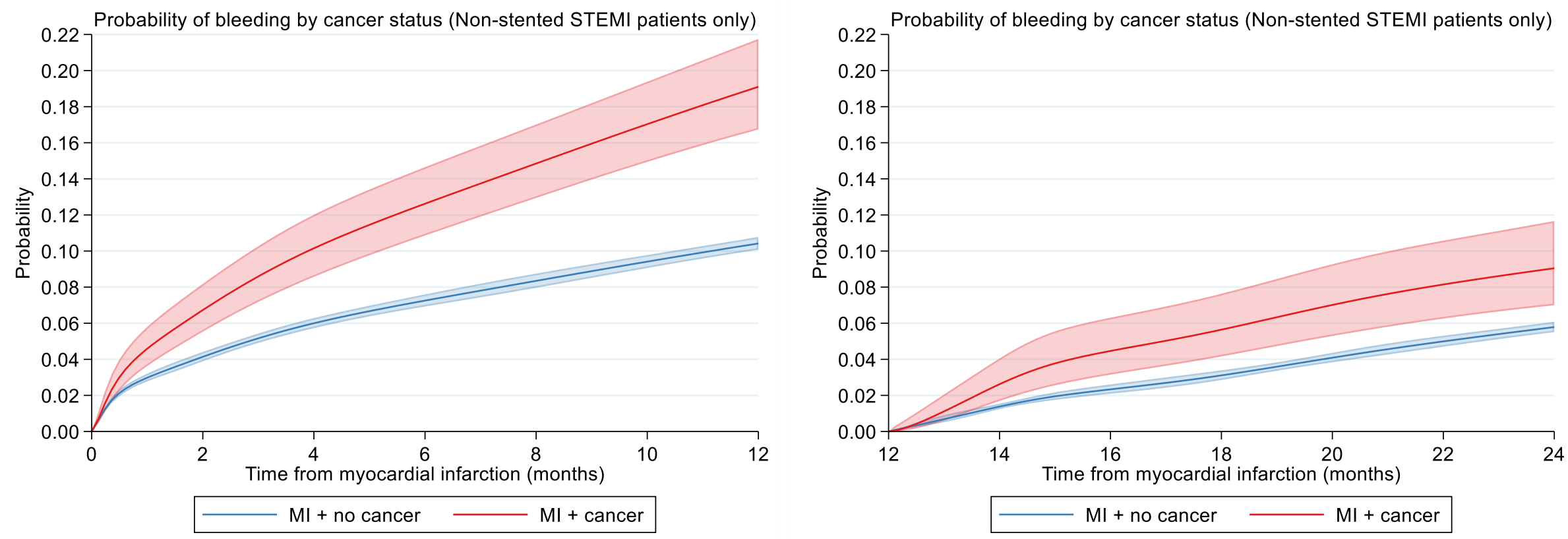

Probability of presentation to hospital for bleeding, comparing cancer patients to non-cancer patients in the first year (left) and second year (right) after myocardial infarction using flexible parametric methods, after inverse probability weighting (non-stented STEMI patients only).

Supplemental Figures 12 & 13 Non-stented NSTEMI

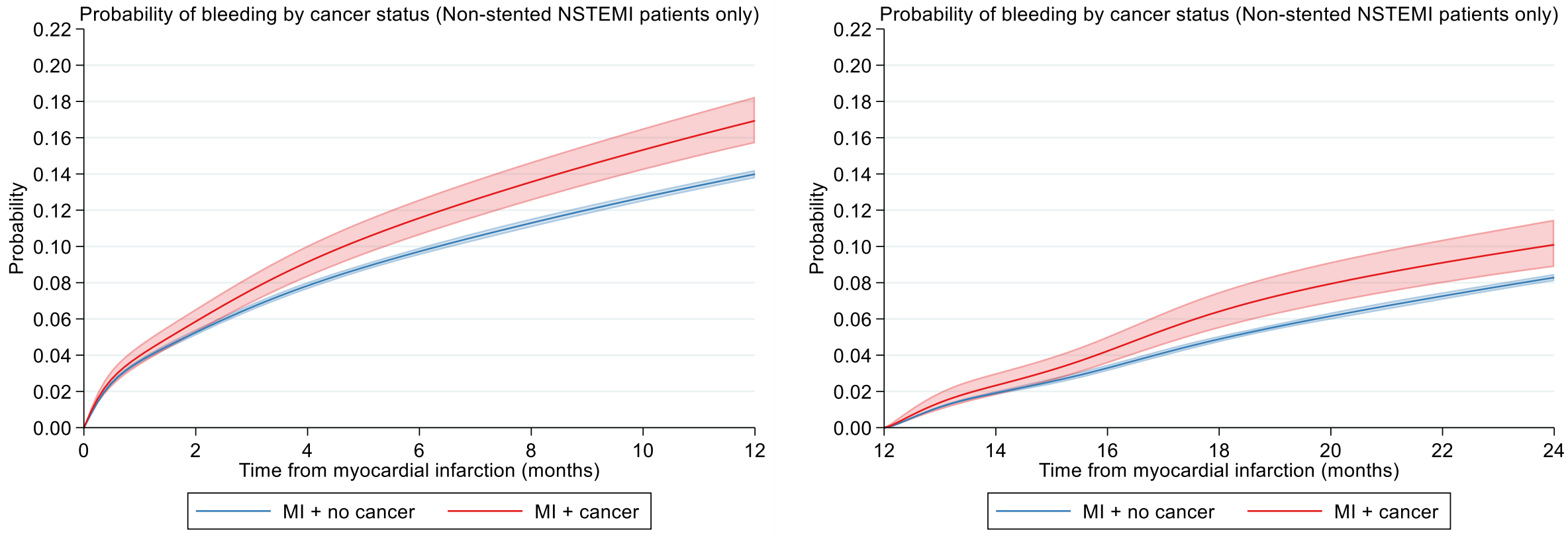

Probability of presentation to hospital for bleeding, comparing cancer patients to non-cancer patients in the first year (left) and second year (right) after myocardial infarction using flexible parametric methods, after inverse probability weighting (non-stented NSTEMI patients only).

Supplemental Figures 14 & 15 Colorectal cancer

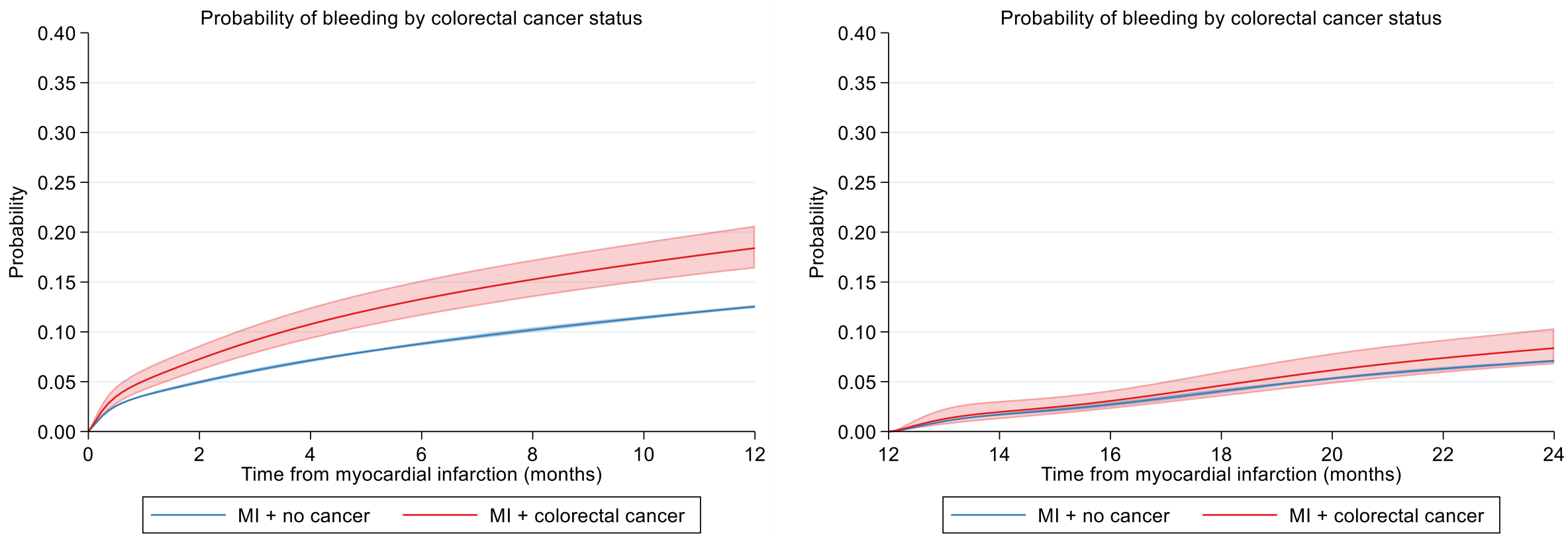

Probability of presentation to hospital for bleeding, comparing colorectal cancer patients to non-cancer patients in the first year (left) and second year (right) after myocardial infarction using flexible parametric methods, after inverse probability weighting.

Supplemental Figures 16 & 17 Lung Cancer

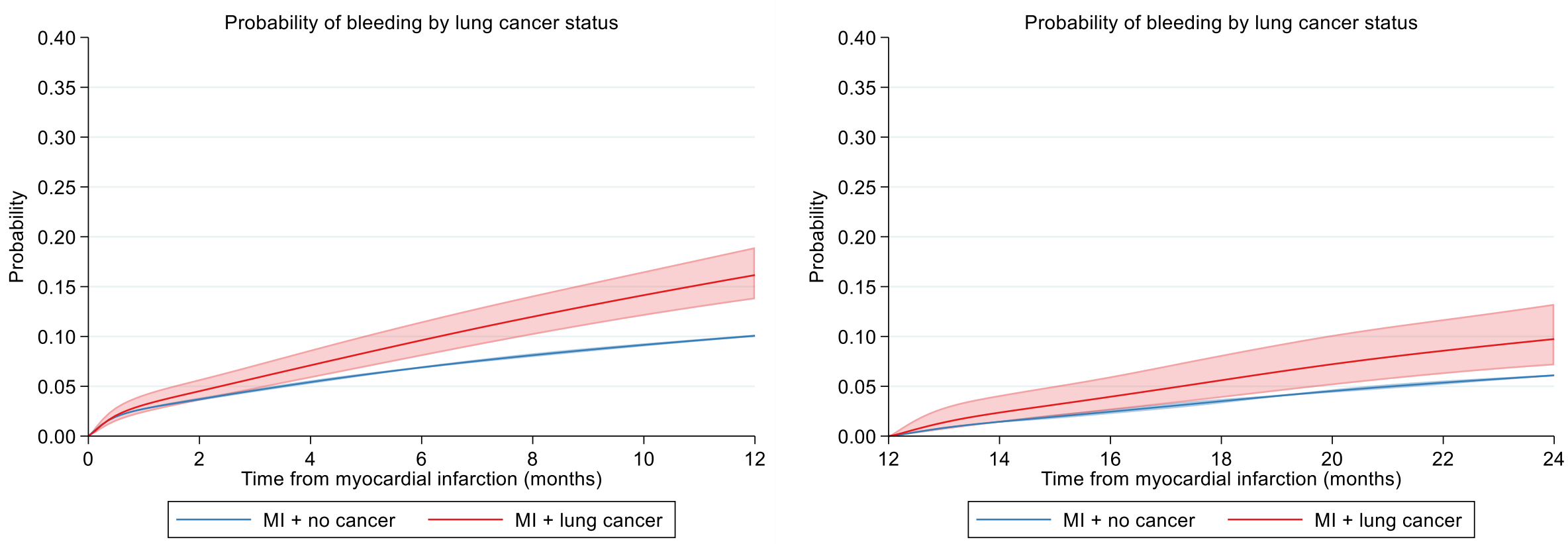

Probability of presentation to hospital for bleeding, comparing lung cancer patients to non-cancer patients in the first year (left) and second year (right) after myocardial infarction using flexible parametric methods, after inverse probability weighting.

Supplemental Figures 18 & 19 Prostate Cancer

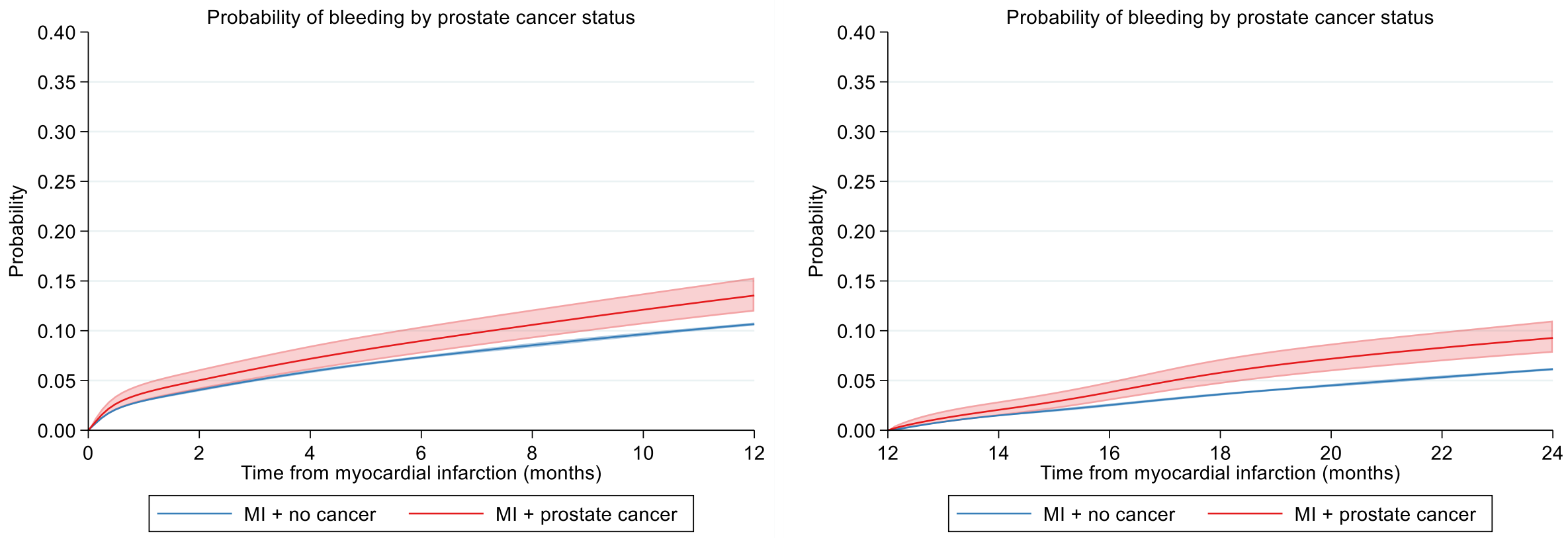

Probability of presentation to hospital for bleeding, comparing prostate cancer patients to non-cancer patients in the first year (left) and second year (right) after myocardial infarction using flexible parametric methods, after inverse probability weighting.

Supplemental Figures 20 & 21 Breast Cancer

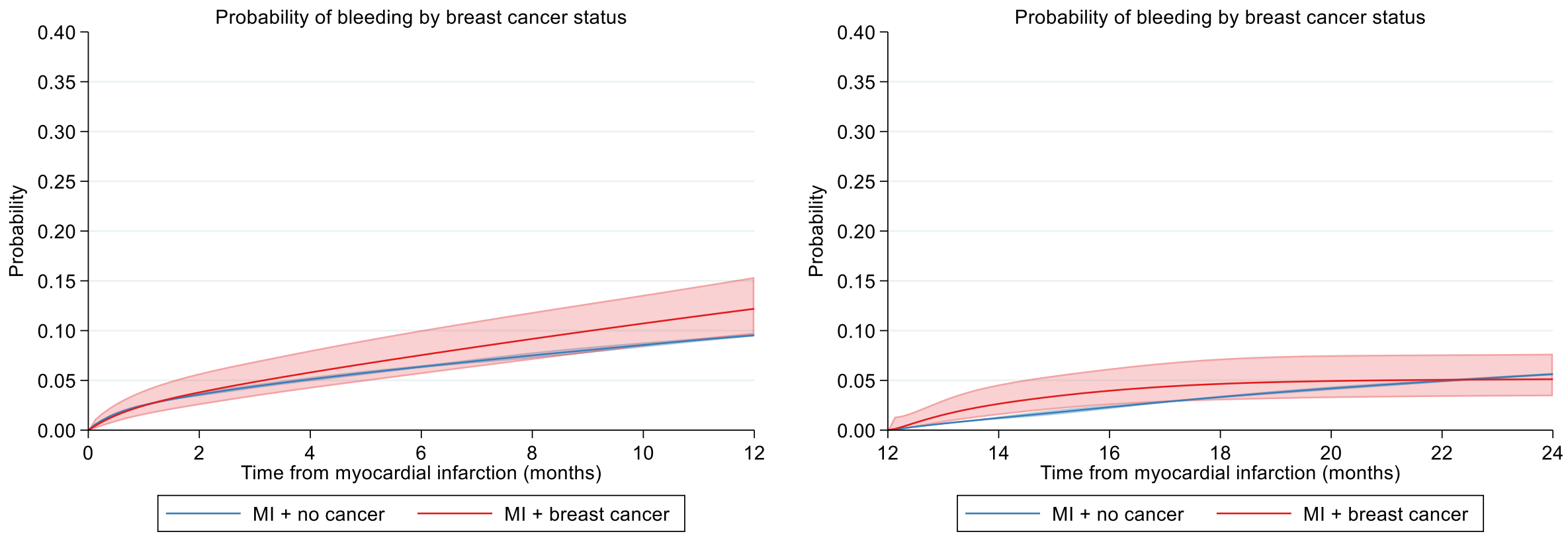

Probability of presentation to hospital for bleeding, comparing breast cancer patients to non-cancer patients in the first year (left) and second year (right) after myocardial infarction using flexible parametric methods, after inverse probability weighting.

Supplemental Figures 22 & 23 Bladder Cancer

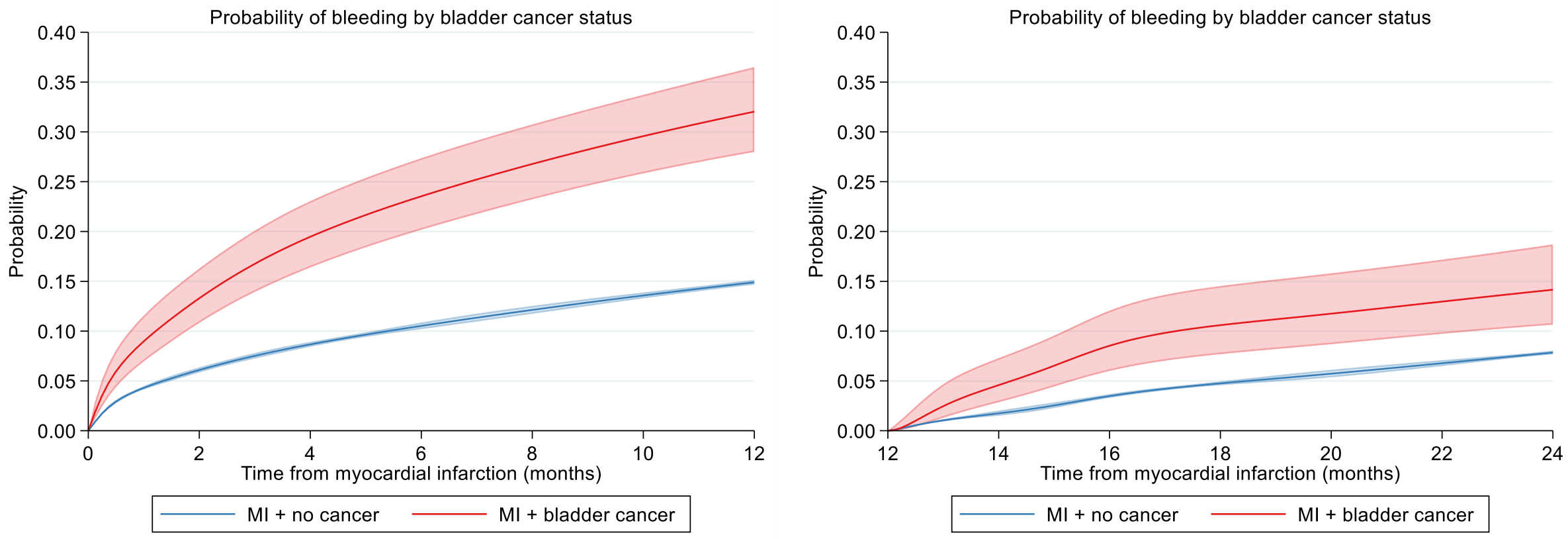

Probability of presentation to hospital for bleeding, comparing bladder cancer patients to non-cancer patients in the first year (left) and second year (right) after myocardial infarction using flexible parametric methods, after inverse probability weighting.

Sensitivity Analysis

Supplemental Figure 24

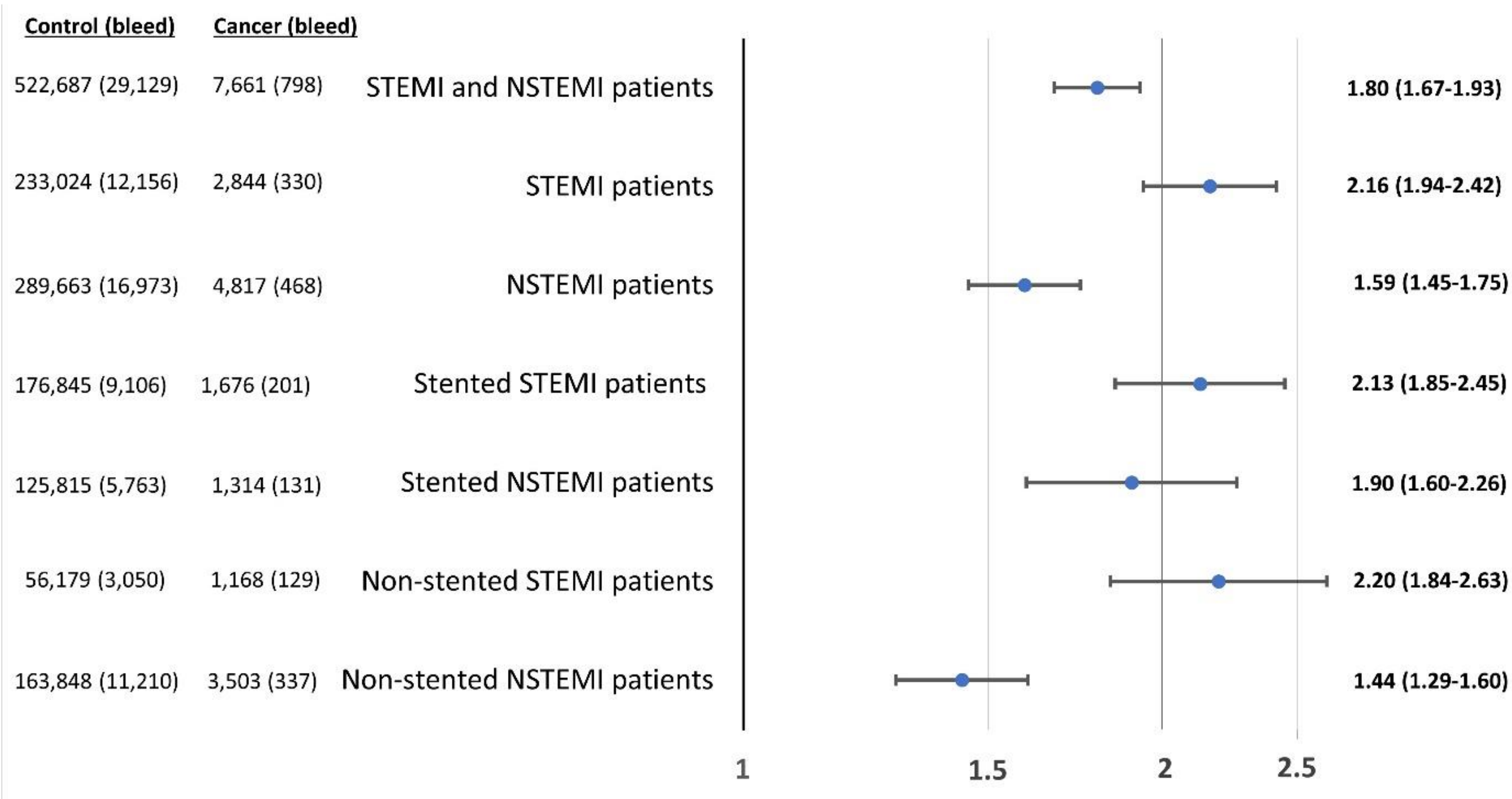

Forest plot demonstrating hazard ratios for presentation to hospital for bleeding comparing cancer patients to non-cancer patients in the first year after myocardial infarction stratified by type of MI and coronary stent status, excluding patients who have had a previous bleed as a sensitivity analysis (after inverse probability weighting, log scale).

Supplemental Figure 25

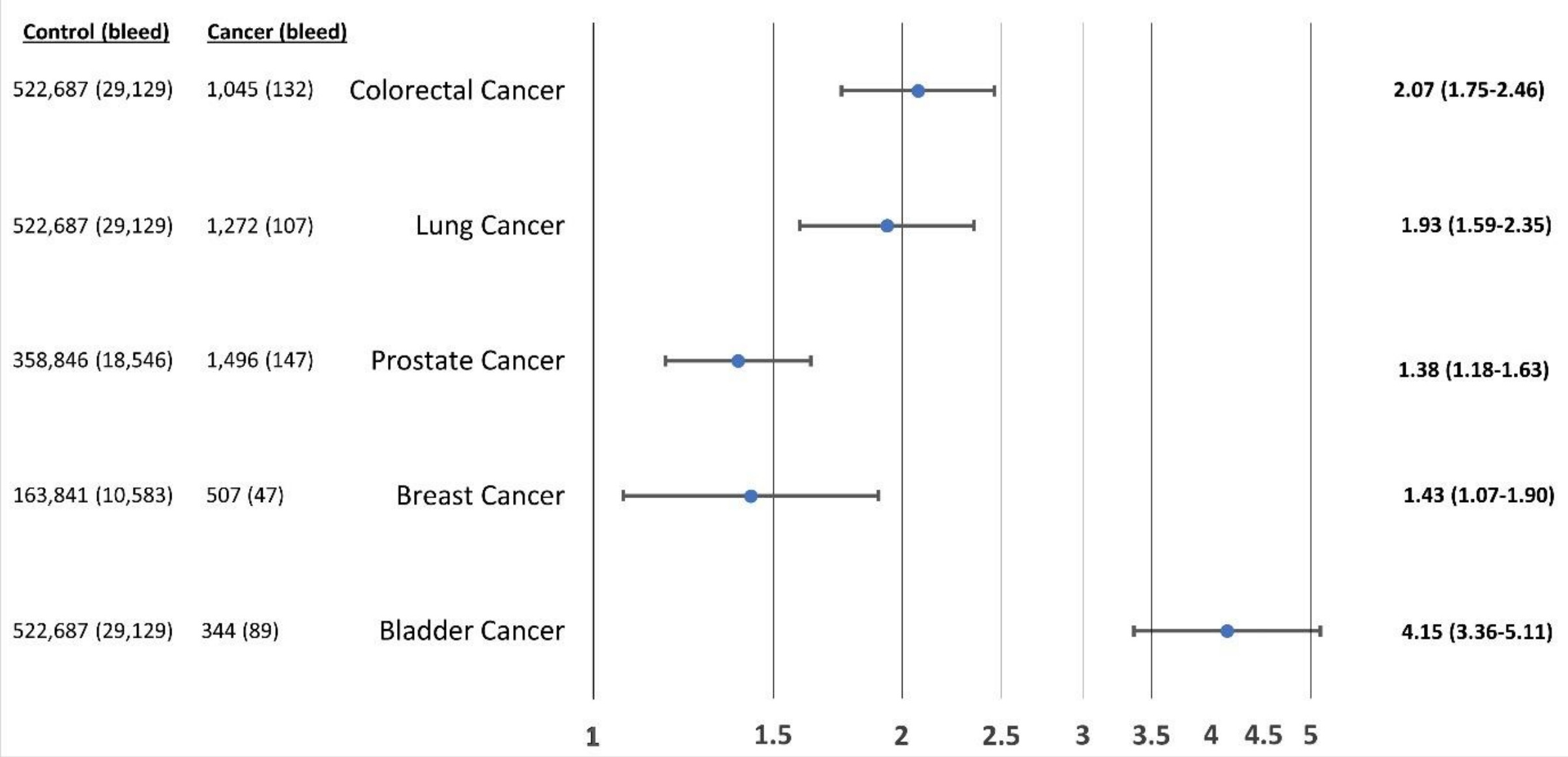

Forest plot demonstrating hazard ratios for presentation to hospital for bleeding comparing cancer patients to non-cancer patients in the first year after myocardial infarction stratified by cancer type, excluding patients who have had a previous bleed as a sensitivity analysis (after inverse probability weighting, log scale).

Supplemental Figure 26

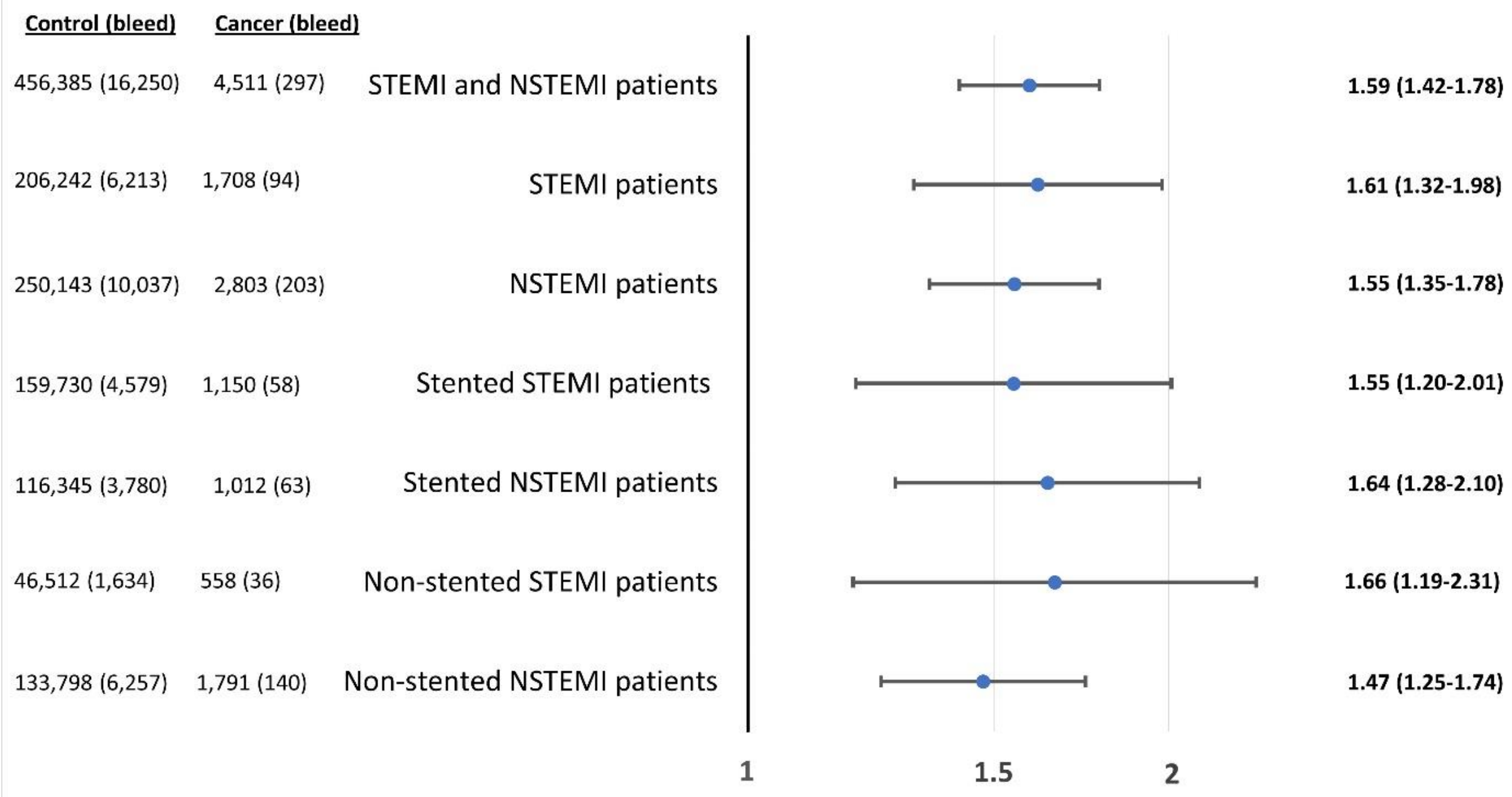

Forest plot demonstrating hazard ratios for presentation to hospital for bleeding comparing cancer patients to non-cancer patients in the second year after myocardial infarction stratified by type of MI and coronary stent status, excluding patients who have had a previous bleed as a sensitivity analysis (after inverse probability weighting, log scale).

Supplemental Figure 27

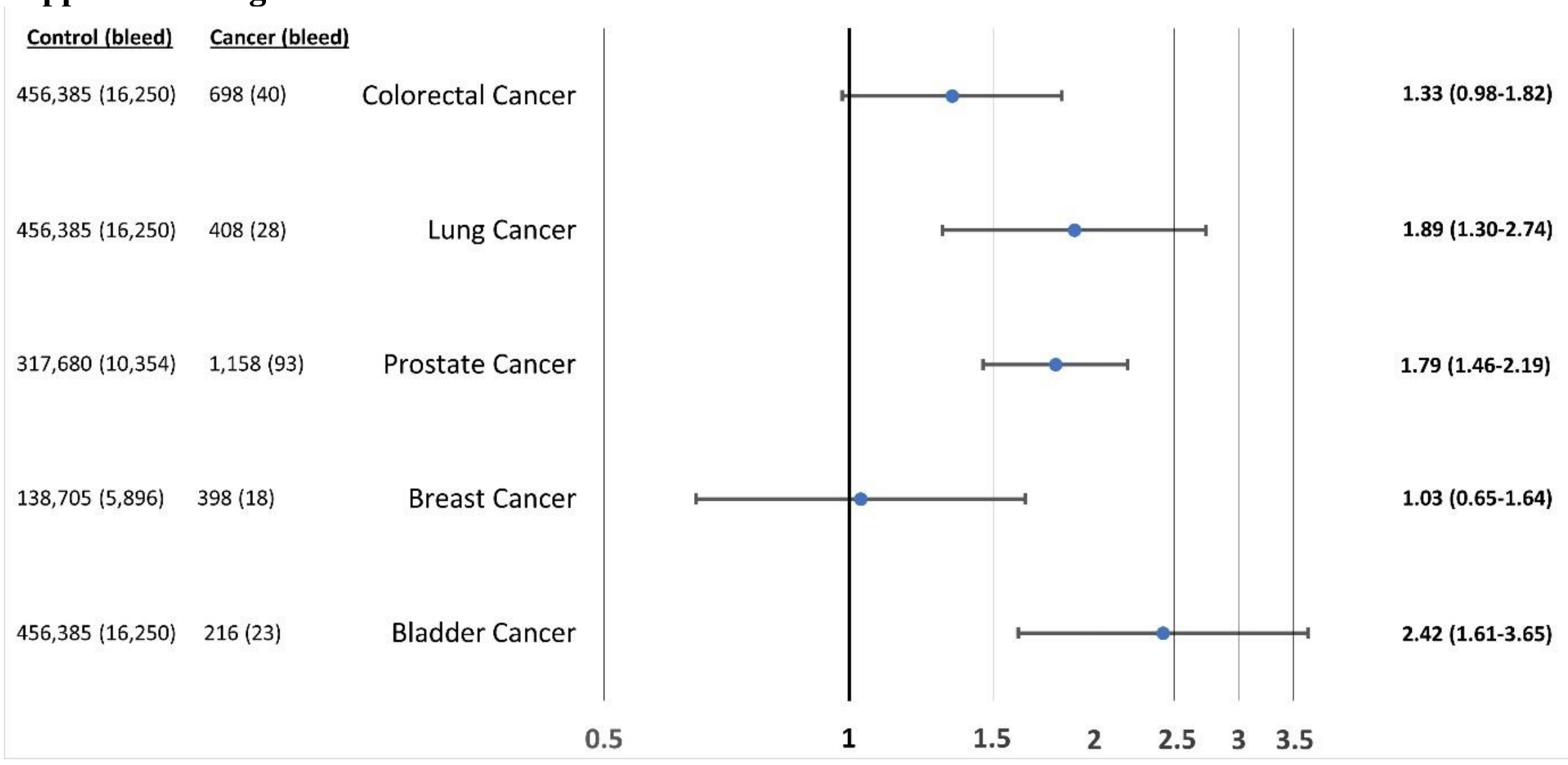

Forest plot demonstrating hazard ratios for presentation to hospital for bleeding comparing cancer patients to non-cancer patients in the second year after myocardial infarction stratified by cancer type, excluding patients who have had a previous bleed as a sensitivity analysis (after inverse probability weighting, log scale).

Supplemental Figures for flexible parametric methods, comparing the first and second year after MI, excluding patients who have had a previous bleed as a sensitivity analysis.

Supplemental Figures 28 & 29

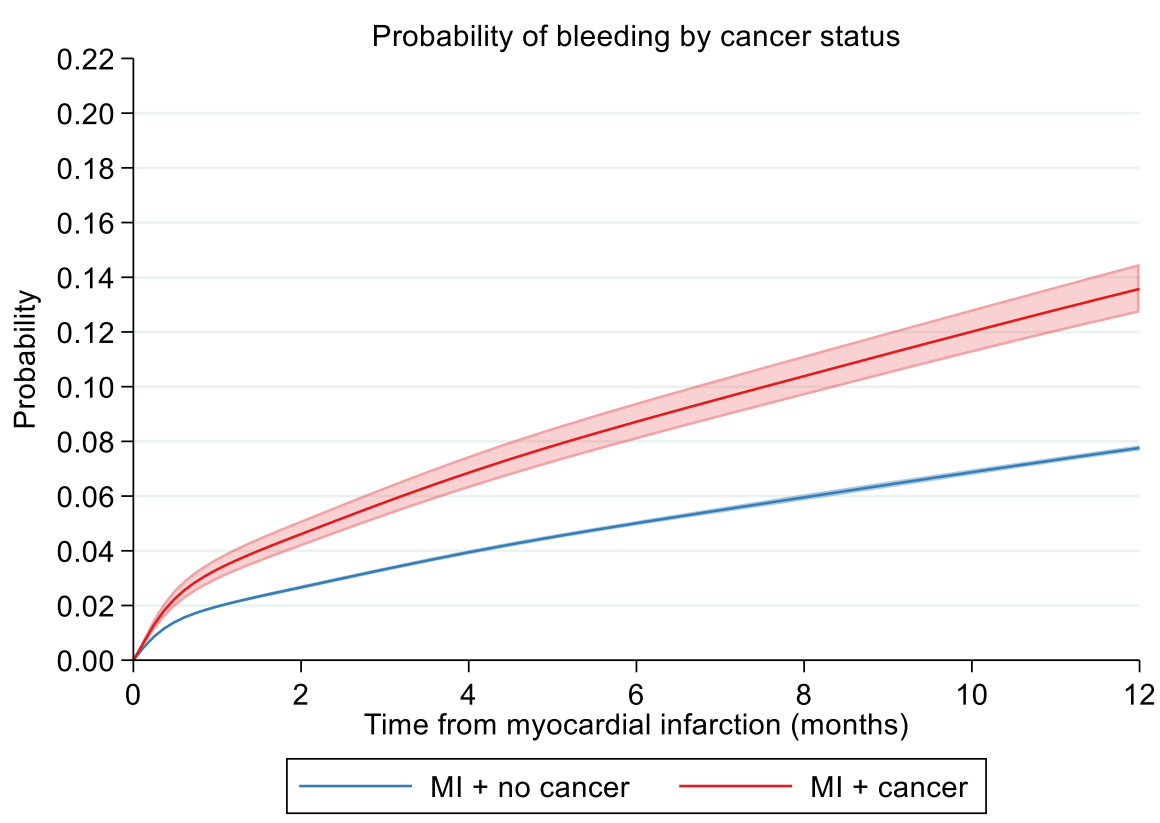

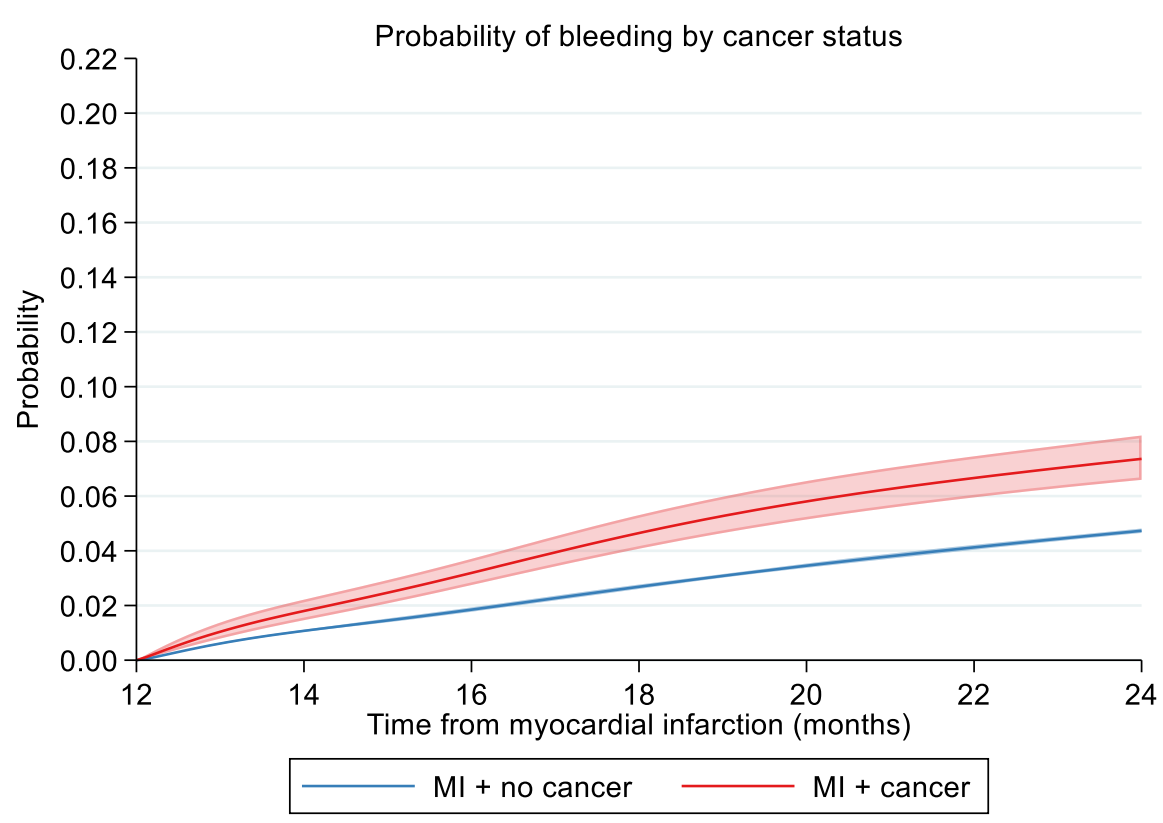

Probability of presentation to hospital for bleeding, comparing cancer patients to non-cancer patients in the first year (left) and second year (right) after myocardial infarction using flexible parametric methods, after inverse probability weighting, excluding patients who have had a previous bleed as a sensitivity analysis.

Supplemental Figures 30 & 31 STEMI only

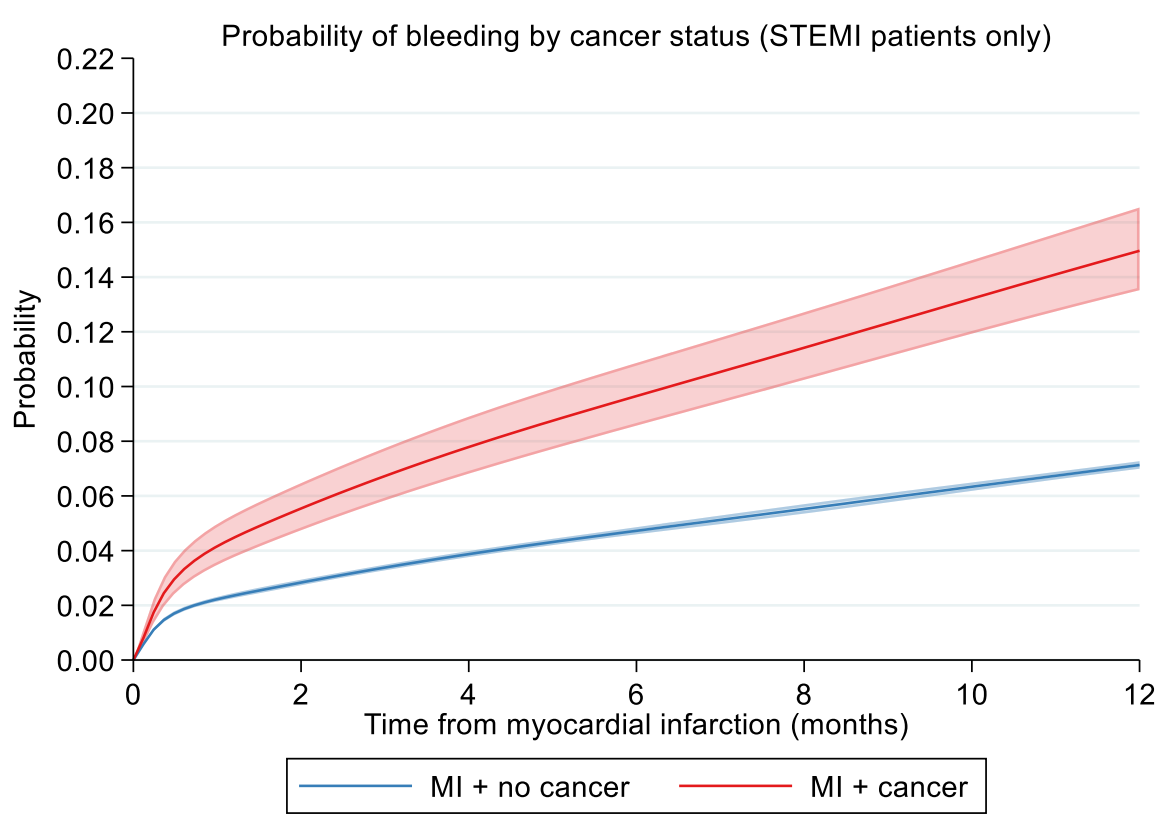

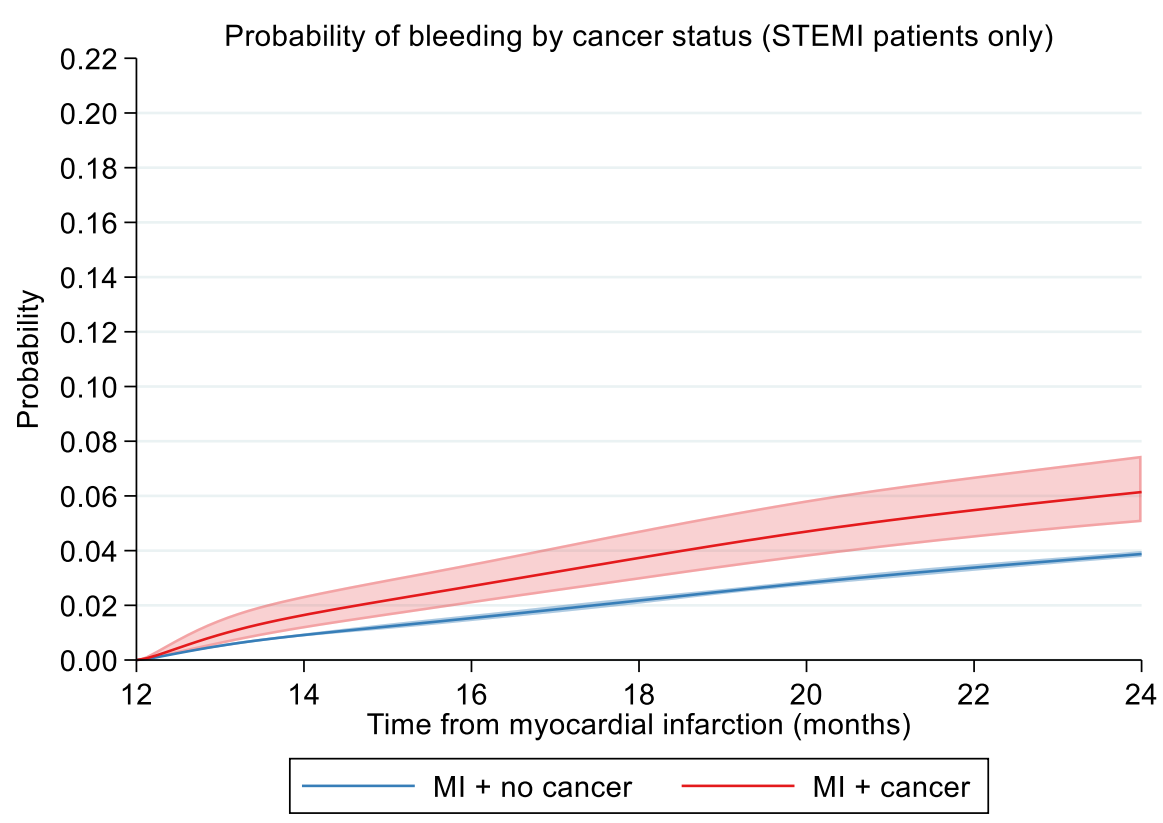

Probability of presentation to hospital for bleeding, comparing cancer patients to non-cancer patients in the first year (left) and second year (right) after myocardial infarction using flexible parametric methods, after inverse probability weighting, excluding patients who have had a previous bleed as a sensitivity analysis (STEMI patients only).

Supplemental Figures 32 & 33 NSTEMI Only

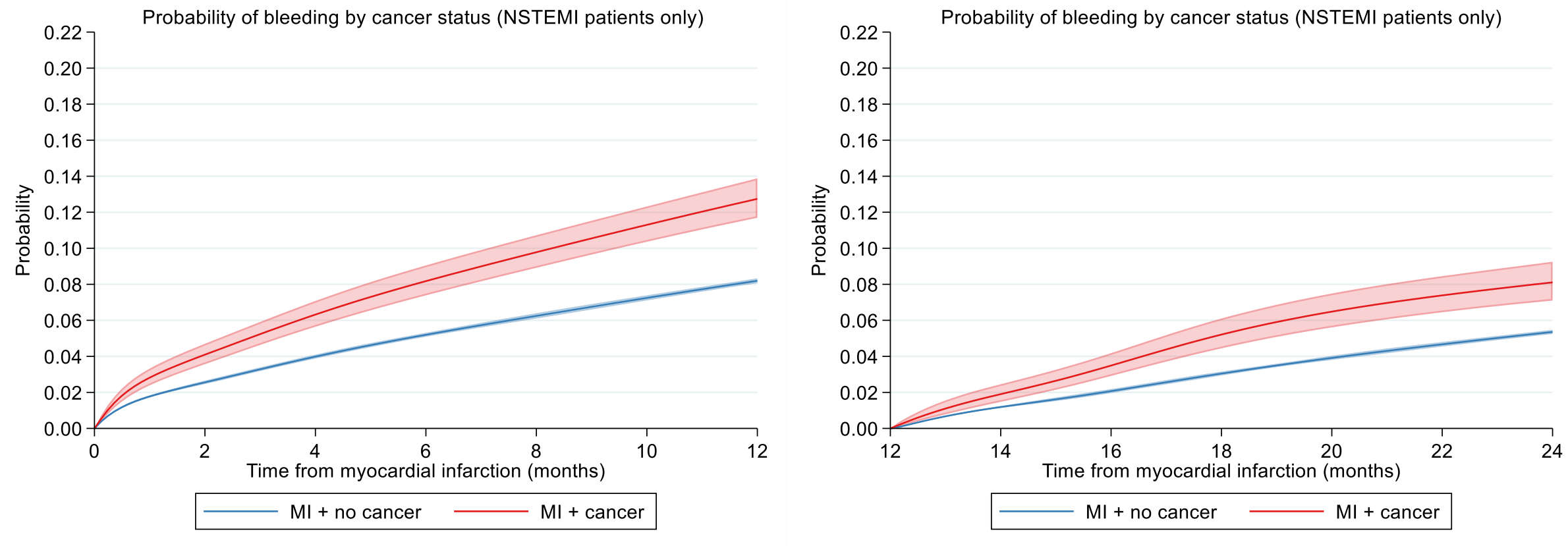

Probability of presentation to hospital for bleeding, comparing cancer patients to non-cancer patients in the first year (left) and second year (right) after myocardial infarction using flexible parametric methods, after inverse probability weighting, excluding patients who have had a previous bleed as a sensitivity analysis (NSTEMI patients only).

Supplemental Figures 34 & 35 Stented STEMI

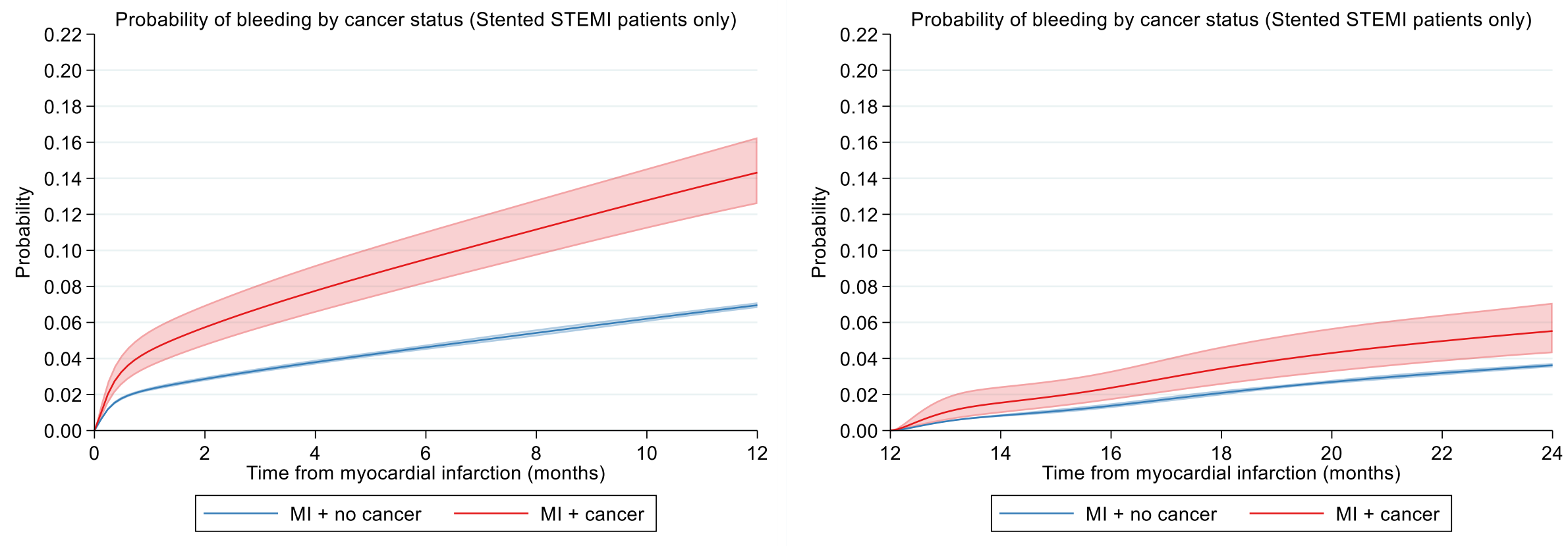

Probability of presentation to hospital for bleeding, comparing cancer patients to non-cancer patients in the first year (left) and second year (right) after myocardial infarction using flexible parametric methods, after inverse probability weighting, excluding patients who have had a previous bleed as a sensitivity analysis (Stented STEMI patients only).

Supplemental Figures 36 & 37 Stented NSTEMI

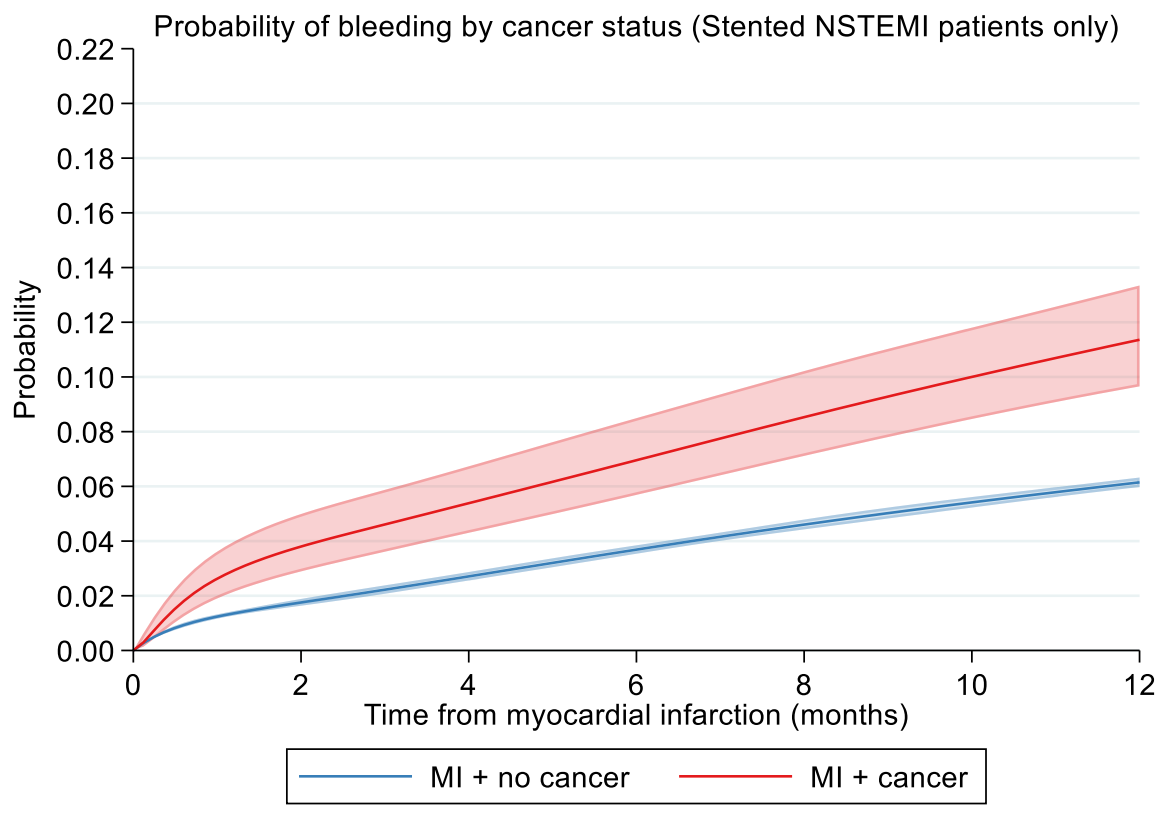

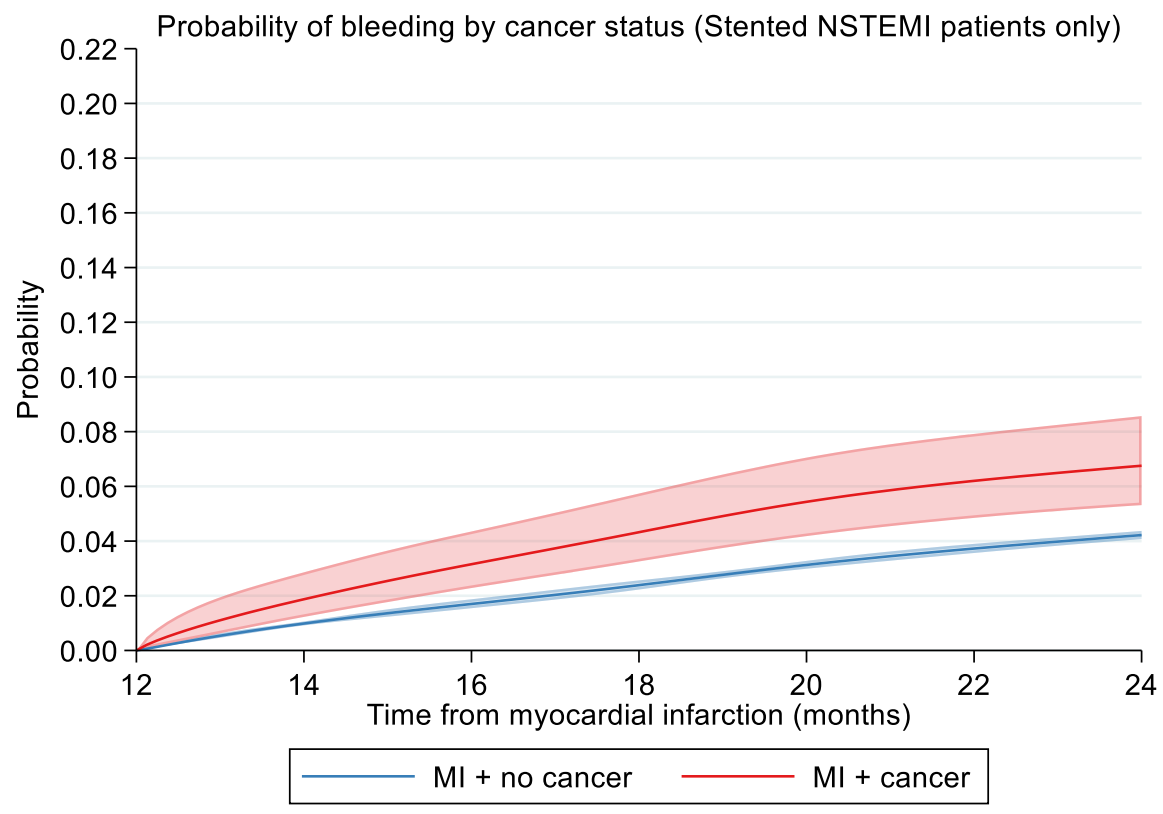

Probability of presentation to hospital for bleeding, comparing cancer patients to non-cancer patients in the first year (left) and second year (right) after myocardial infarction using flexible parametric methods, after inverse probability weighting, excluding patients who have had a previous bleed as a sensitivity analysis (Stented NSTEMI patients only).

Supplemental Figures 38 & 39 Non-stented STEMI

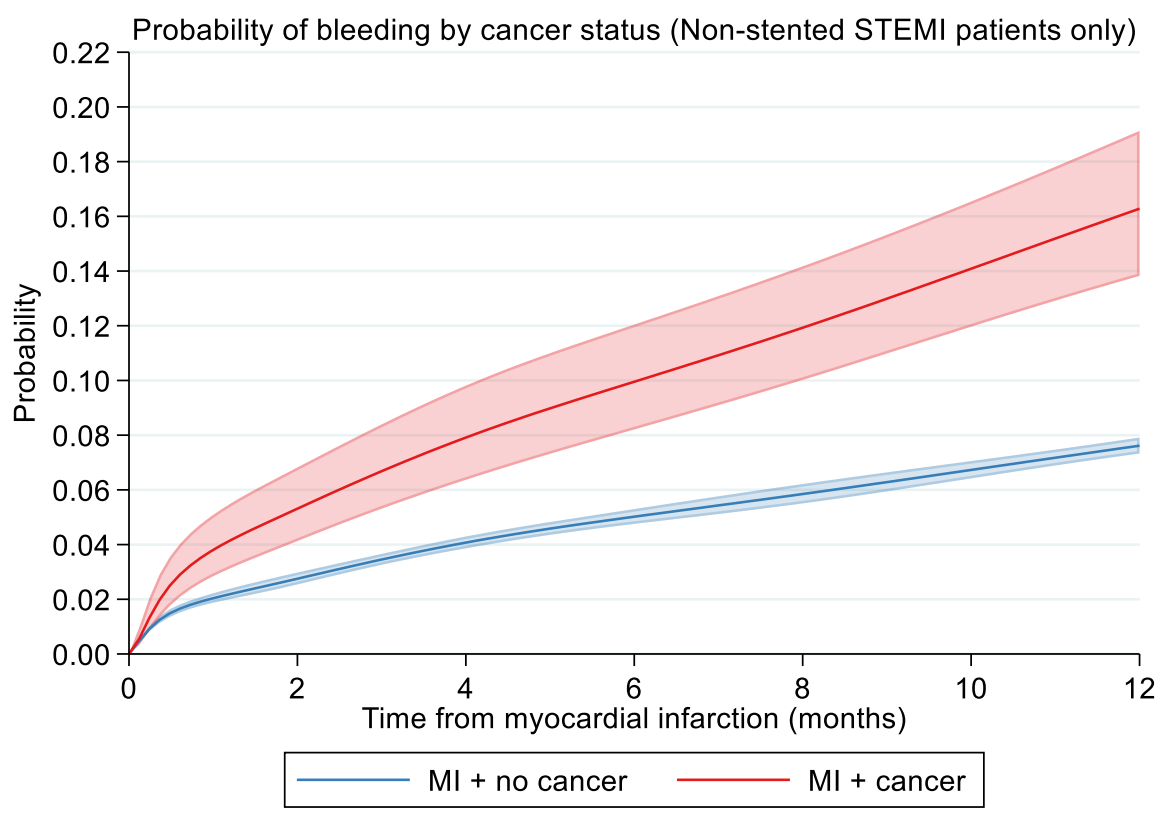

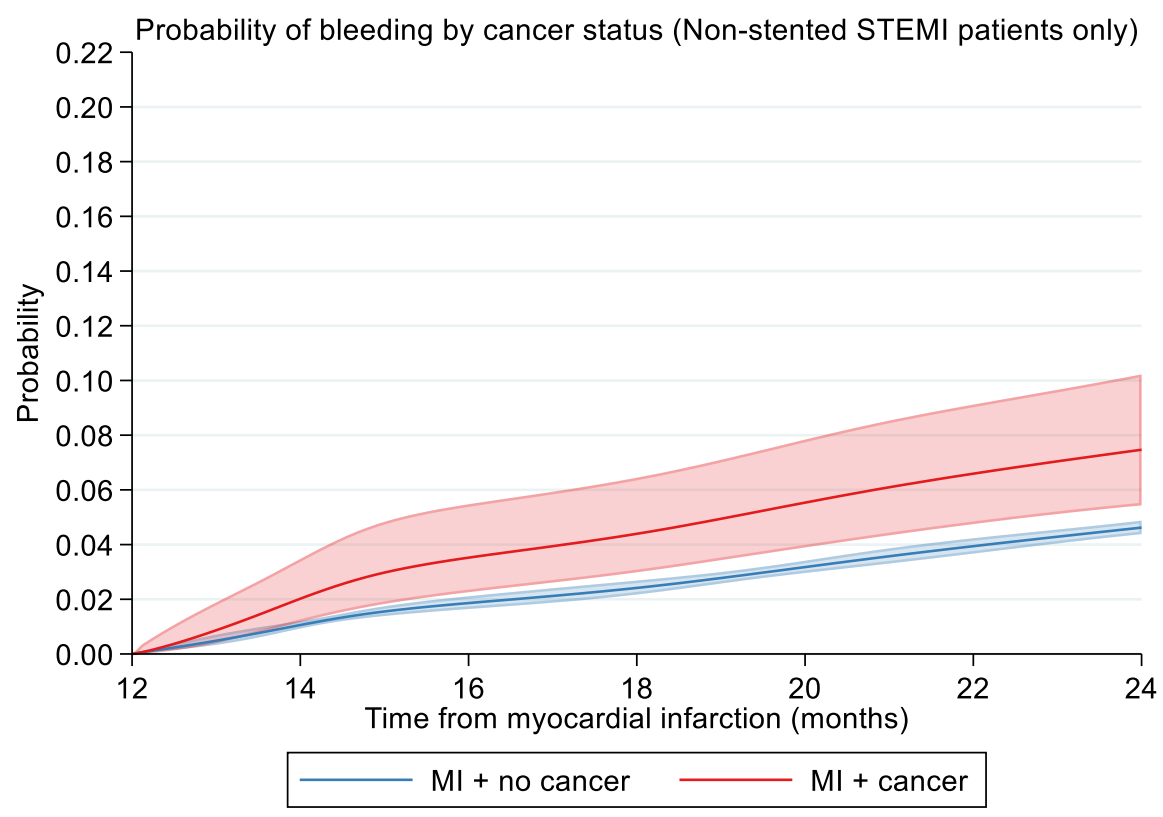

Probability of presentation to hospital for bleeding, comparing cancer patients to non-cancer patients in the first year (left) and second year (right) after myocardial infarction using flexible parametric methods, after inverse probability weighting, excluding patients who have had a previous bleed as a sensitivity analysis (non-stented STEMI patients only).

Supplemental Figures 40 & 41 Non-stented NSTEMI

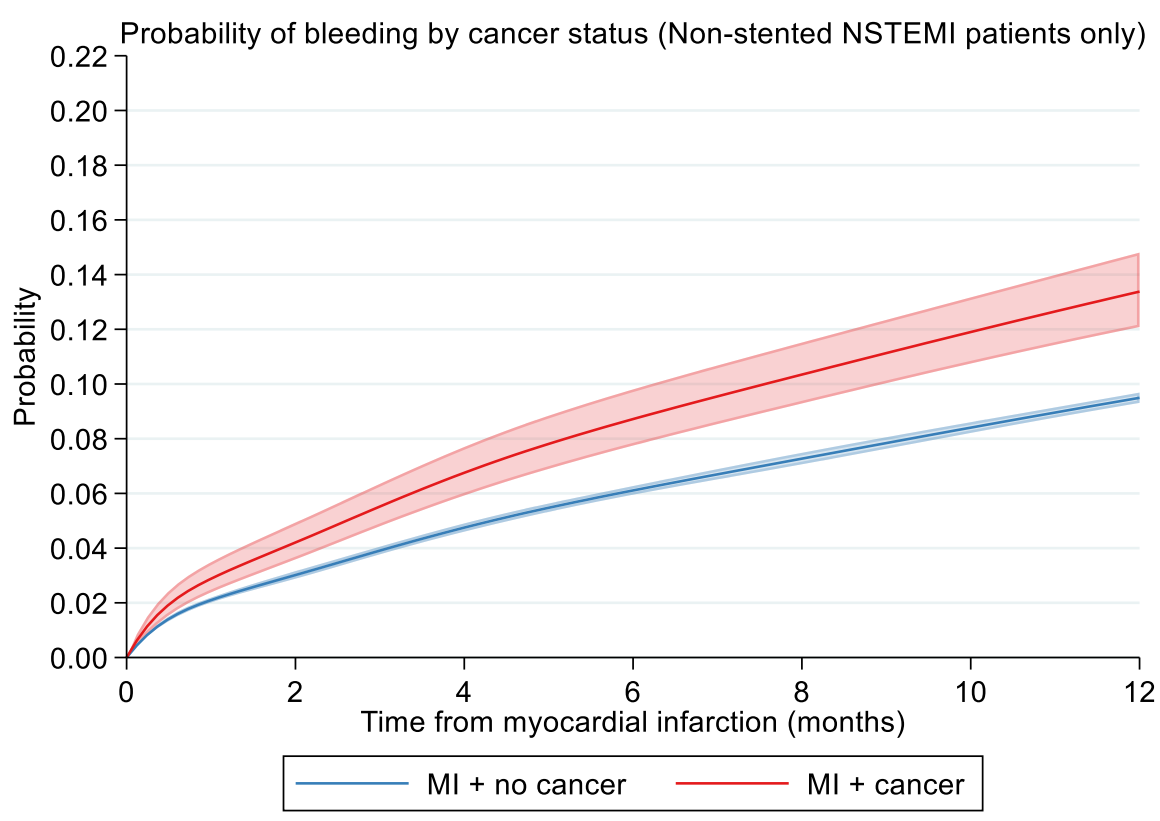

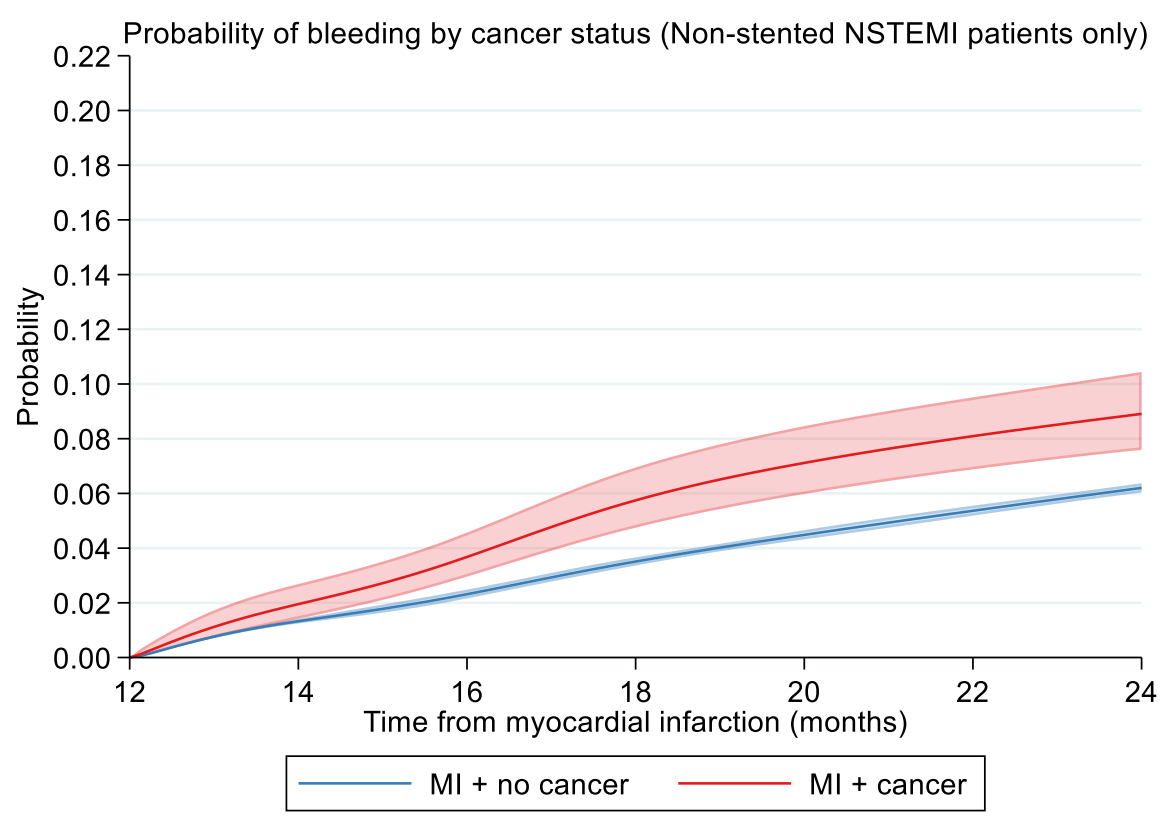

Probability of presentation to hospital for bleeding, comparing cancer patients to non-cancer patients in the first year (left) and second year (right) after myocardial infarction using flexible parametric methods, after inverse probability weighting, excluding patients who have had a previous bleed as a sensitivity analysis (non-stented NSTEMI patients only).

Supplemental Figures 42 & 43 Colorectal cancer

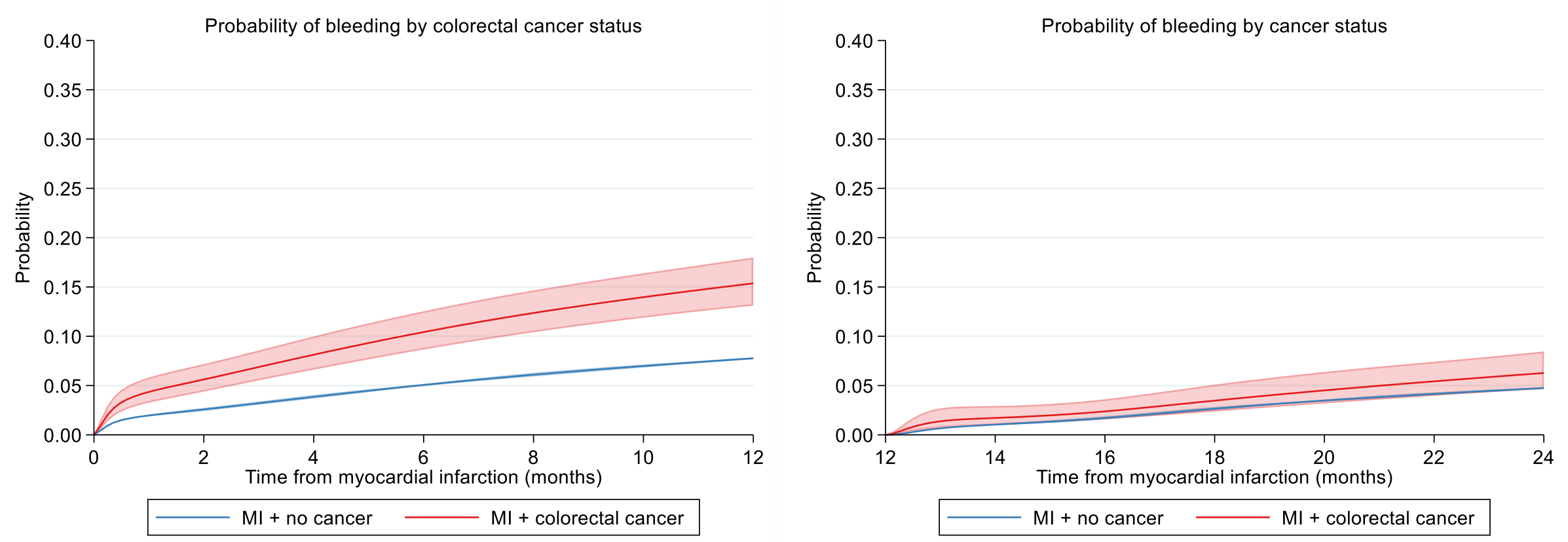

Probability of presentation to hospital for bleeding, comparing colorectal cancer patients to non-cancer patients in the first year (left) and second year (right) after myocardial infarction using flexible parametric methods, after inverse probability weighting, excluding patients who have had a previous bleed as a sensitivity analysis.

Supplemental Figures 44 & 45 Lung Cancer

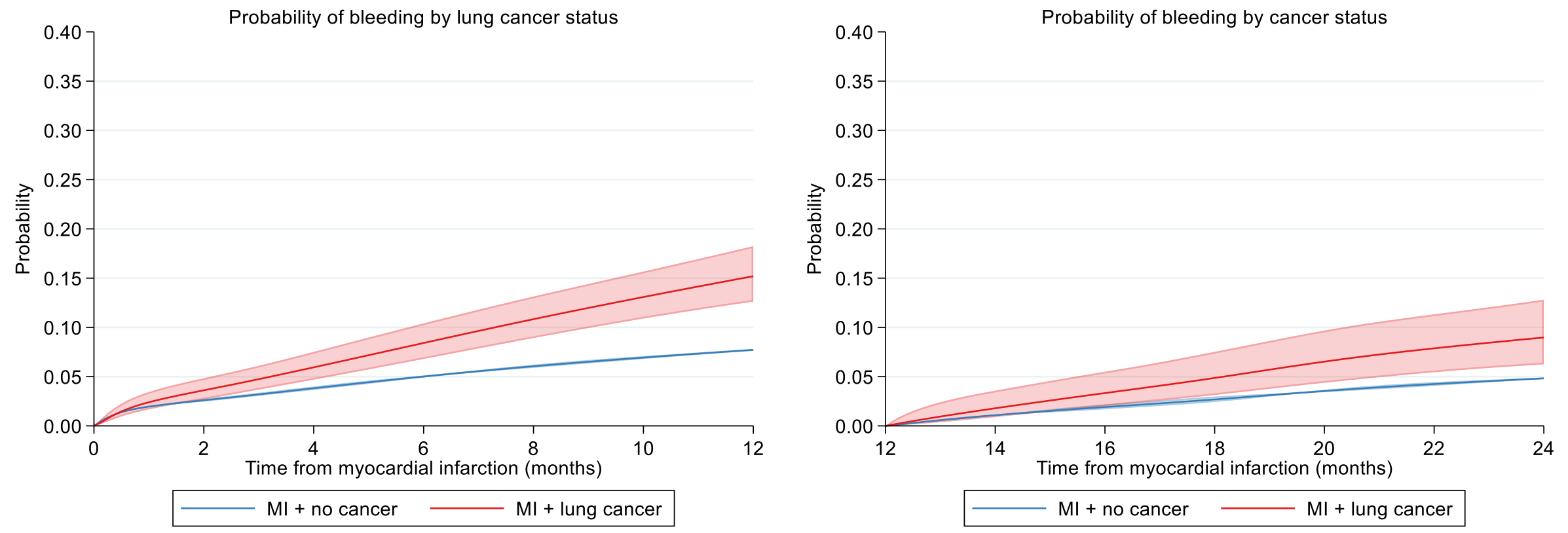

Probability of presentation to hospital for bleeding, comparing lung cancer patients to non-cancer patients in the first year (left) and second year (right) after myocardial infarction using flexible parametric methods, after inverse probability weighting, excluding patients who have had a previous bleed as a sensitivity analysis.

Supplemental Figures 46 & 47 Prostate Cancer

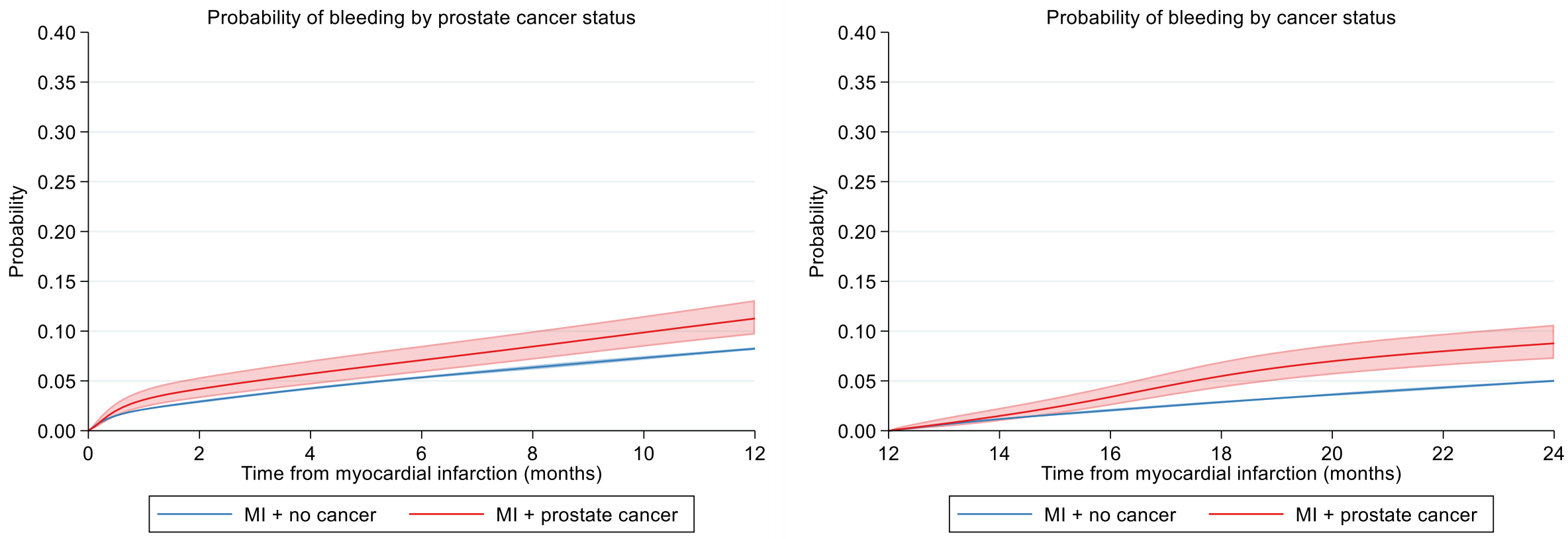

Probability of presentation to hospital for bleeding, comparing prostate cancer patients to non-cancer patients in the first year (left) and second year (right) after myocardial infarction using flexible parametric methods, after inverse probability weighting, excluding patients who have had a previous bleed as a sensitivity analysis.

Supplemental Figures 48 & 49 Breast Cancer

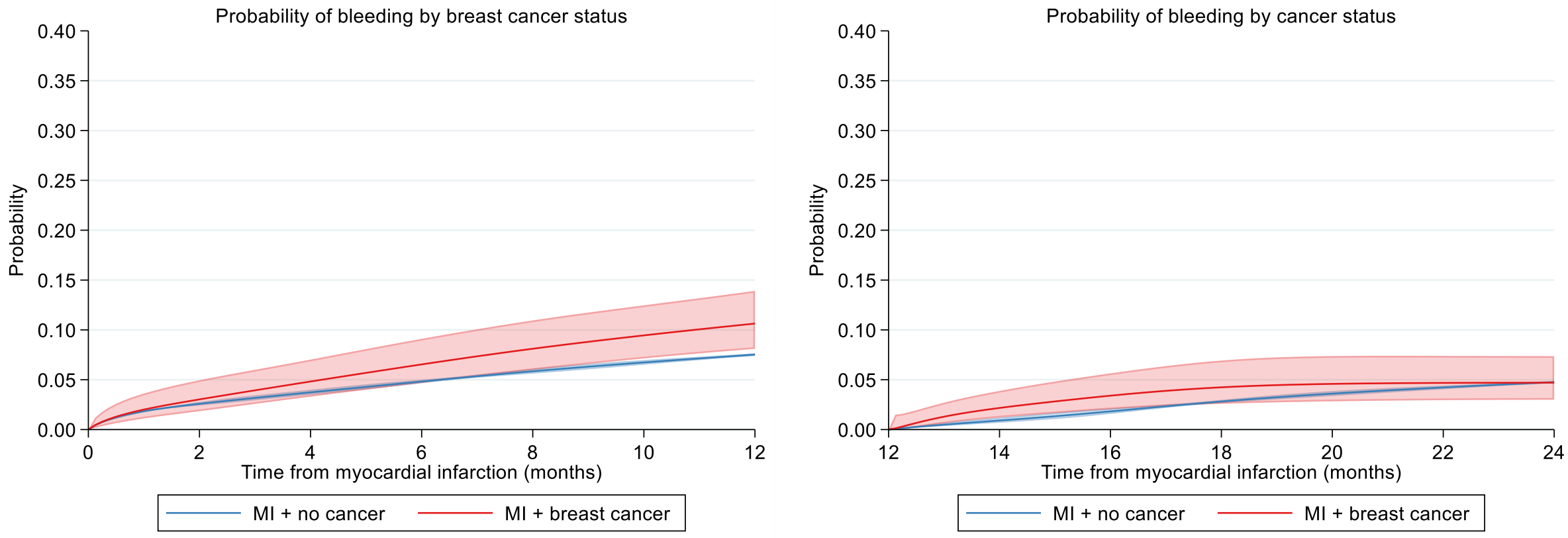

Probability of presentation to hospital for bleeding, comparing breast cancer patients to non-cancer patients in the first year (left) and second year (right) after myocardial infarction using flexible parametric methods, after inverse probability weighting, excluding patients who have had a previous bleed as a sensitivity analysis.

Supplemental Figures 50 & 51 Bladder Cancer

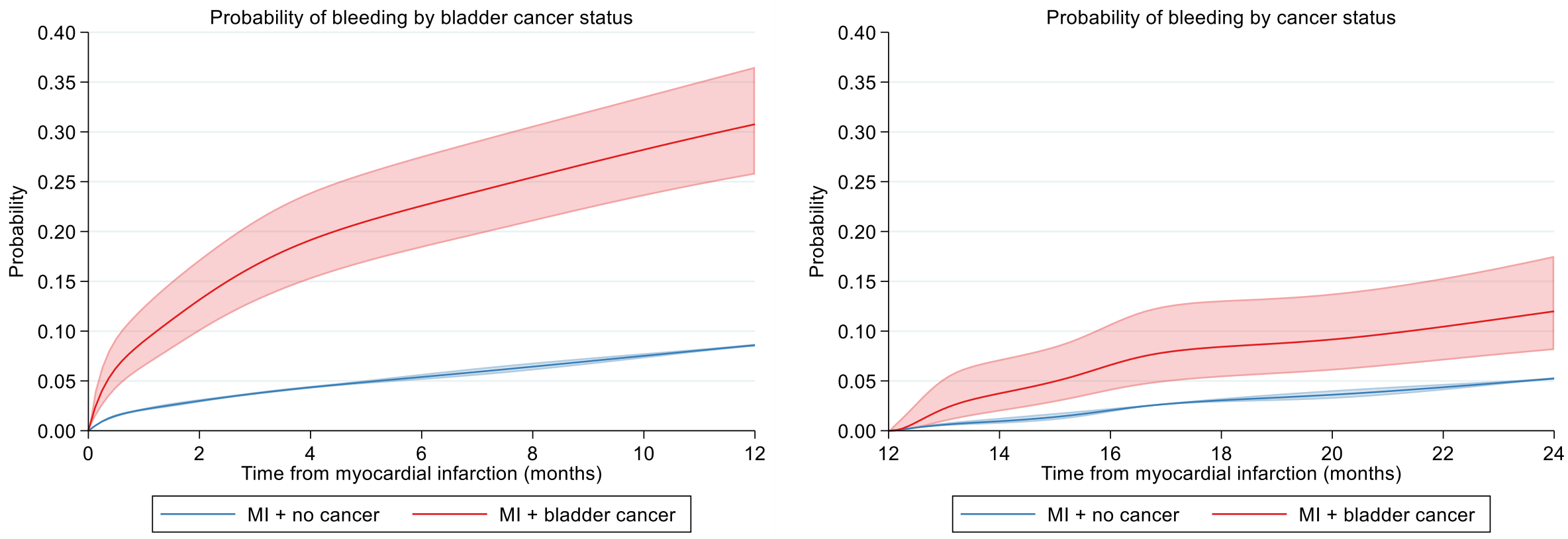

Probability of presentation to hospital for bleeding, comparing bladder cancer patients to non-cancer patients in the first year (left) and second year (right) after myocardial infarction using flexible parametric methods, after inverse probability weighting, excluding patients who have had a previous bleed as a sensitivity analysis

Supplemental Figure 52

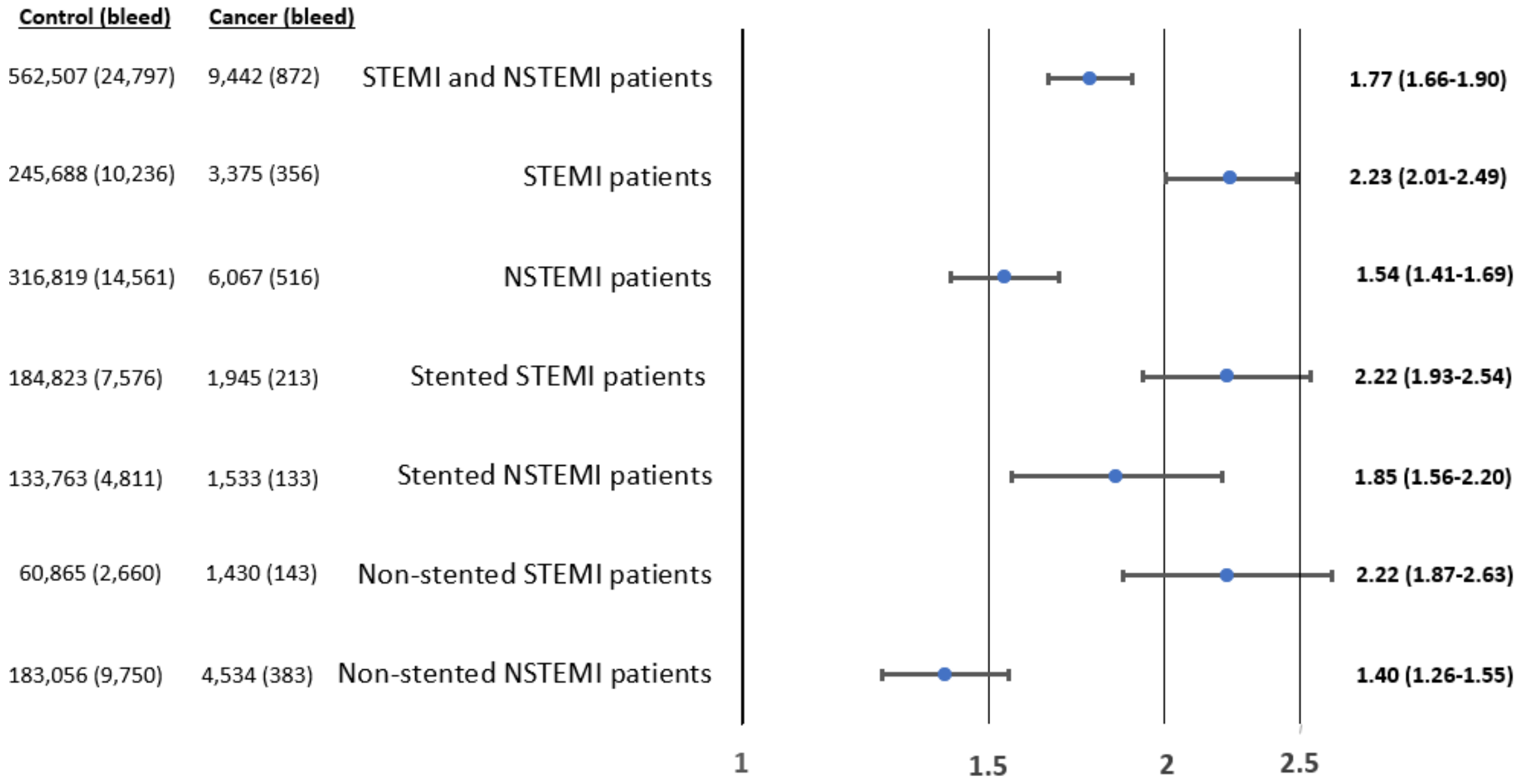

Forest plot demonstrating hazard ratios for presentation to hospital for bleeding comparing cancer patients to non-cancer patients in the first year after myocardial infarction stratified by type of MI and coronary stent status, excluding patients with iron deficiency anaemia as a sensitivity analysis (after inverse probability weighting, log scale).

Supplemental Figure 53

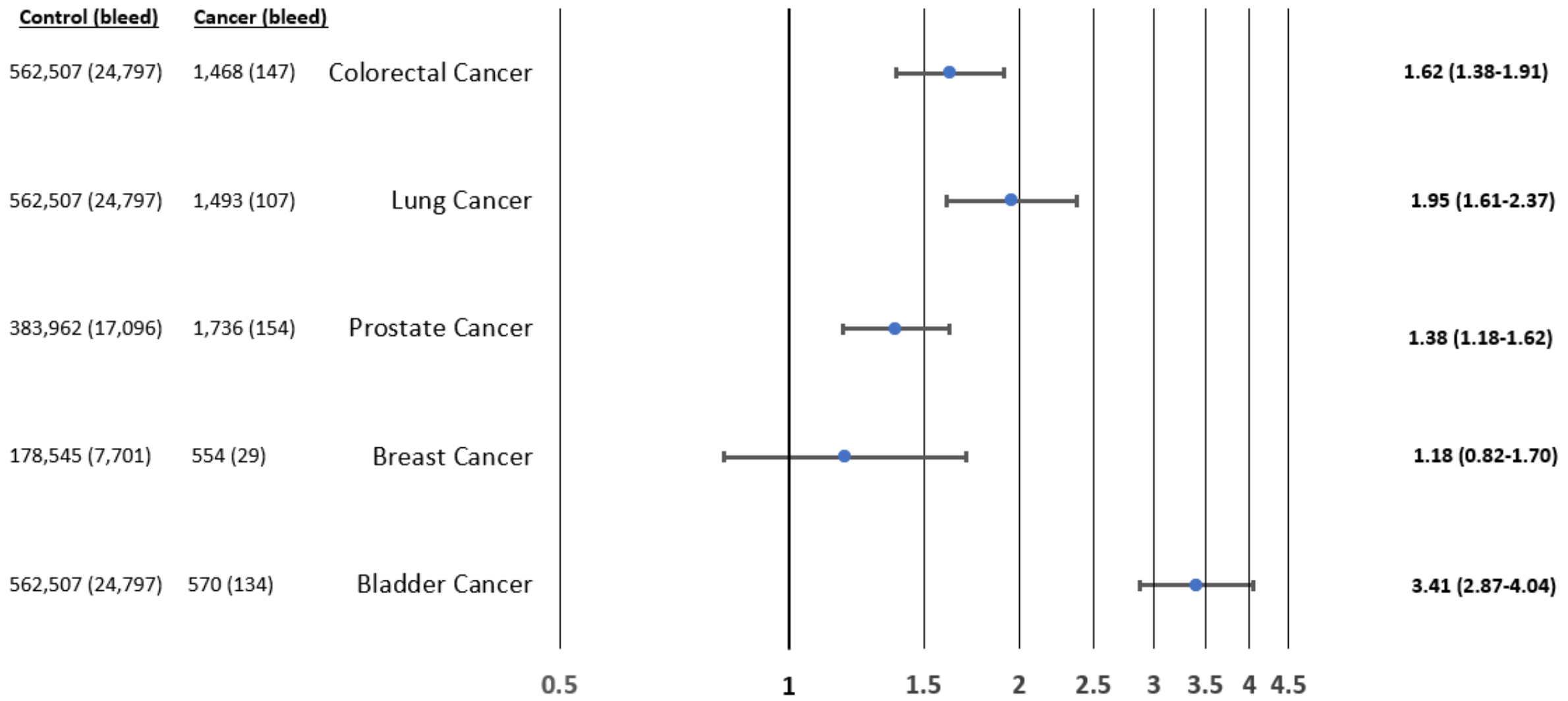

Forest plot demonstrating hazard ratios for presentation to hospital for bleeding comparing cancer patients to non-cancer patients in the first year after myocardial infarction stratified by cancer type, excluding patients with iron deficiency anaemia as a sensitivity analysis (after inverse probability weighting, log scale).

Supplemental Figure 54 Bootstrapped Confidence Intervals

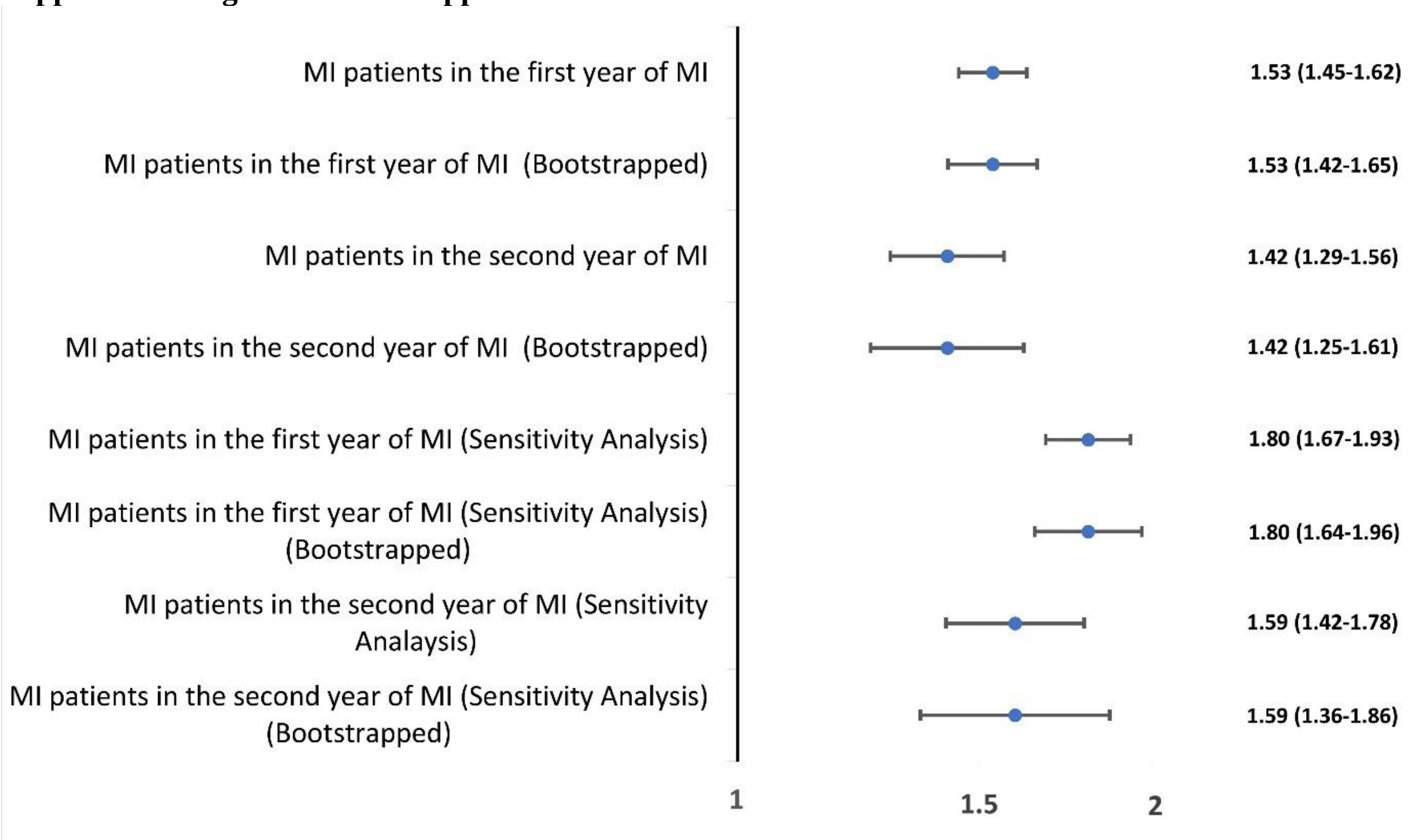

Supplemental Figure 52: Forest plot demonstrating hazard ratios for presentation to hospital for bleeding comparing cancer patients to non-cancer patients, comparing results from the original analysis against respective bootstrapped results (after inverse probability weighting, log scale).

References

1. Battisti NML, Welch CA, Sweeting M, et al. Prevalence of Cardiovascular Disease in Patients With Potentially Curable Malignancies: A National Registry Dataset Analysis. *JACC CardioOncology*. 2022;4(2):238-253. doi:10.1016/j.jaccao.2022.03.004
2. Jun M, James MT, Manns BJ, et al. The association between kidney function and major bleeding in older adults with atrial fibrillation starting warfarin treatment: population based observational study. *BMJ (Clinical research ed)*. 2015;350doi:10.1136/bmj.h246
